# Environmental surveillance and spatio-temporal analysis of *Legionella spp*. in a region of northeastern Italy (2002–2017)

**DOI:** 10.1371/journal.pone.0218687

**Published:** 2019-07-09

**Authors:** Antonella Felice, Marinella Franchi, Stefano De Martin, Nicola Vitacolonna, Lucilla Iacumin, Marcello Civilini

**Affiliations:** 1 Laboratory of Microbiology, ARPA - Regional Agency for Environmental Protection Friuli Venezia Giulia, Udine, Italy; 2 Department of Mathematics, Computer Science and Physics, University of Udine, Udine, Italy; 3 Department of Agriculture, Food, Environmental and Animal Sciences, University of Udine, Udine, Italy; Purdue University, UNITED STATES

## Abstract

*Legionella spp*. are considered an important cause of potentially preventable morbidity and mortality, making environmental surveillance a crucial component of risk assessment plans. In this work, 20,319 water samples were collected in 3,983 environmental surveys during a 16-year period by ARPA, the Regional Agency for Environmental Protection, Friuli Venezia Giulia, and the results were studied to better understand the diffusion mechanisms of *Legionella*. The data showed a strong seasonal signal, a prevalence of *L. pneumophila* serogroup 2–15 in most environments (63% of positive samples), a prevalence of *L. pneumophila* serogroup 1 in swimming pool-associated environments (82% of positive samples), a persistent presence of *Legionella* in hospitals and a recurrent presence of *Legionella* in other facilities such as hotels, possibly years after interventions, highlighting the difficulty of eradicating the bacteria. Retrospective spatio-temporal analyses on geocoded historical data were carried out with SaTScan using an ordinal model with risk as a covariate to identify potential clusters with an excess of cases in the higher-risk categories. Although no outbreaks occurred during the period of study, such analyses identified spatially restricted zones with unusual contamination, which sometimes were also areas in which several surveys triggered by notifications of clinical cases were performed. Simulations of periodic prospective analyses permitted the assessment of the efficacy of the method in early detection of such clusters. The proposed method may be a useful tool in environmental surveillance, prevention and control of *Legionella*.

## Introduction

*L. pneumophila* is the main causative agent of legionellosis—pulmonary morbid forms normally are acquired by inhalation of aerosols containing the bacterium—which occurs in various pathologies among which pneumonia is the most severe form [1]. Legionellosis is considered a water-borne disease [2] and *Legionella* is among the main emerging pathogenic bacteria of the last fifty years [3]. As part of the normal aquatic microflora, *Legionella* can be found both in natural and man-made aquatic environments, surviving free, in biofilms or as a parasite of protozoa [4–6]. Consequently, *Legionella* can colonize the anthropic habitat through the water distribution system if favorable conditions to its development are present [7, 8]. Some factors, such as excessive exploitation of sources of water supply, presence of industrial discharges and purification plants, deterioration of water supply systems may be controlled or prevented [9], but others, such as people’s changing habits (e.g., the increased use of the shower instead of the bathtub, the diffusion of air conditioning systems causing aerosolization of the water or the use of heated drinking water with warm water reservoirs in houses) have less predictable outcomes [10–12]; it may therefore be quite difficult to implement adequate control measures. Demographic changes leading to an aging population and a growing number of susceptible individuals, the adoption of new behaviors, such as considerable propensity for the use of air-conditioning systems, and the presence of wellness centers and similar facilities causing water aerosolization are among the factors that determine an increase of hydrodiffused epidemics in industrialized countries and affect the incidence of legionellosis [9, 13].

Given their resistance to potabilization treatments, legionellae may contaminate water supply and distribution systems. Even if damaged or injured by such treatments, legionellae can survive in a condition defined as *viable but not culturable* (*VBNC*), which prevents the growth of such microorganisms in common culture media and causing analyses to produce false negative results. *Legionella*’s VBNC cells represent a risk to public health since such undetectable cells may be active when they reach the distribution network where they often find low concentrations of chlorine and the presence of nutrients that allow their growth [7, 9, 14]. *L. pneumophila* by itself does not prefer human as the host [10, 15, 16], but new lifestyles and environmental niches created by humans have led legionellosis to be an emerging risk for public health because the diagnosis and treatment of infections are not always quickly performed [17]. Furthermore, current water treatments might lead to selective pressure on the pathogens, which are able to develop a wide range of survival strategies [9, 18–20].

In recent years, the spread of epidemics related to water consumption in industrialized countries, due for example to devices producing aerosols, spas, etc…, has led to greater attention to issues related to the presence of pathogenic microorganisms in treated water resources [21–23]. The difficulty in controlling the diffusion of *L. pneumophila* depends on many factors related to the ubiquity of the bacterium in the environment and its ability to persist in colonized environments and spread through aerosols [18, 19]. In absence of cases, preventive actions can take on fundamental importance to avoid epidemic outbreaks. Currently, control strategies for nosocomial structures and/or others where host subjects are susceptible to the disease are based on preventive actions. Interventions in response to established cases are much more frequent. Given the difficulty in controlling emerging bacteria, it is important to quickly identify the epicenter of a potential epidemic to prevent outbreaks [22, 24, 25].

In Italy, according to the National Surveillance System for Legionnaires’ disease, the number of cases of legionellosis has been progressively increasing, and the same trend was registered worldwide [13, 26]. In 2017, 2,014 cases of legionellosis were notified (of which 1,981 confirmed and 33 probable), equal to 33.2 cases/million inhabitants [26]. The notification of cases of legionellosis to public health authorities in Italy has been mandatory since 1983. The identification of an isolated case of legionellosis triggers investigation procedures with confirmation stages, notification, and epidemiological study geared towards detecting the origin of the exposure to the pathogen. Similarly, two or more cases with supposed common origin within a two-year period prompt a series of verification steps aimed at determining the common source of the infection [27]. In this context, and based on the hypotheses emerging from a descriptive analysis and a targeted anamnesis, environmental surveys on water/air networks and suspicious equipments are carried out. Based on the results of microbiological monitoring, remedial actions on contaminated water plumbing and/or aeraulic pipe are planned, following two main types of criteria: the concentration of *Legionella* and the percentage of positive samples. Evaluation of the risk and disinfection of the infrastructure are mandatorily performed for concentration ranges 101–1,000 and 1,001–10,000 CFUl^−1^, when 30% and 20%, respectively, of the samples are positive. When contamination values are higher than 10,000 CFUl^−1^, positive supply terminals must also be replaced [27].

The national register of legionellosis was established and managed by the “Istituto Superiore di Sanità” (the National Public Health Institute) on the basis of surveillance forms submitted by physicians to the Local Health Units for each Legionnaires’ disease case. Since 2005, a network of reference regional centers responsible for microbiological diagnoses and environmental control of legionellosis within their respective regional areas has been established [27, 28]. ARPA-FVG (“Agenzia Regionale per la Protezione dell’Ambiente del Friuli Venezia Giulia”) is the reference regional center for the Friuli Venezia Giulia region, in northeastern Italy (S1 Fig). ARPA-FVG is accredited in accordance with ISO/IEC 17025:2005 and ISO 11731:2017 standards for *Legionella* testing [29, 30] and carries out its activities following confirmed clinical cases and/or control actions prescribed by the Health Regional Authorities, with the purpose of identifying and preventing possible outbreaks. ARPA-FVG follows the ECDC Legionnaires’ disease surveillance network called European Legionnaires’ Disease Surveillance Network (ELDSNet) for the investigation of travel associated epidemic clusters as well as hospital acquired [31, 32] to highlight the risk factors and interrupt the transmission chain [32]. In Friuli Venezia Giulia, 32 cases of legionellosis were notified in 2017, equal to 26.2 cases/million inhabitants [26].

In this work, historical data about the environmental surveys carried out in the region Friuli Venezia Giulia by ARPA-FVG since 2002 were analyzed for a better understanding of the dispersion patterns of *L. pneumophila* associated with the environment. The main information consisted of time and address of each survey and the results of culture analysis for each sample collected during the survey. A clustering method suitable for this type of highly correlated data was applied and tuned to retrospectively identify periods and areas with frequently considerable contamination levels. The same method was then used to simulate prospective analyses and validate the hypothesis that the same clusters could be detected early. The goal was to provide better insight into this opportunistic pathogen at different geographical scales and to define a predictive epidemiological method suitable to be adopted by the public health authorities of the region in the coming years.

## Materials and methods

### Microbiological examination

ARPA-FVG received water samples collected at various sites by qualified personnel of the Local Health Units. The sampling and analysis protocols were in accordance with the Italian national Guidelines [27] and adhered to international standard directives [30]. Discriminatory selection of presumptive positive colonies was made on *Legionella BCYE Agar Base supplemented with Legionella BCYE Growth Supplement with and without cysteine* (Biolife Italiana s.r.l., Milan, Italy), and those ascribable to the *Legionella* genus were serologically identified using monovalent antisera (Biolife Ref. 271050—*Legionella* rapid latex test Kit). Since 2012, real-time PCR following Annex 6 of the Italian national Guidelines [27] has joined culture methods. The method consists of different phases: sample concentration by filtration of 1 liter of water sample, DNA extraction and purification by Aquadien DNA Extraction and Purification Kit (Bio-Rad Laboratories s.r.l., Milan, Italy) and amplification by qualitative real-time PCR (IQ-Check screen *L. pneumophila* Kit BIORAD Cat. 3578105). Quantification of DNA was performed according to ISO/TS 12869:2012 [33]. Correlation was checked by means of linear regression computed positive results expressed in colony forming units (CFUl^−1^) and genome units (GU l^−1^) from the analyzed samples.

### Surveys and environmental sampling

A total of 20,319 water samples were collected through 3,983 environmental surveys during a period of 5,785 days from 19 February 2002 to 21 December 2017. Two types of environmental surveys were conducted: *non-clinical surveys*, routinely performed as part of the regional environmental surveillance plan; and *follow-up* (or *clinical*) surveys, targeted at verifying the presence of the bacterium, mostly in residential buildings, as a consequence of a reported clinical case of legionellosis. Each sample was classified into one of 8 categories (private, tourism, elderly, health, recreation, school, military, other) based on the available description of the surveyed site (Table 1). Other variables extracted from the annotations included the serogroups of the samples that resulted positive, information about the sampling procedure, and a qualitative description of the water circuit where each sample was taken from. The sampling procedure was either *quantitative*, i.e., referring to samples harvested after flaming or disinfecting at the outlet point and after running the water for a while in order to verify the hygienic conditions of the system, or *instantaneous*, i.e., referring to samples harvested without flaming and letting the water run, in order to simulate the possible exposure of the user. The water circuit was annotated as either *cold* or *hot*, depending on whether the sample was collected from a cold water system (or a system without hot water recirculation) or from a hot water system [27]. Each sample was assigned a *risk level* based on its contamination, according to the following conventional classification [27, 32]: *none* (<100 CFUl^−1^), *low* (100 ≤ CFUl^−1^ ≤ 1,000), *medium* (1,000 ≤ CFUl^−1^ ≤ 10,000) and *high* (>10,000 CFUl^−1^) risk. The limit of detection (LOD) was 100 CFUl^−1^. Each survey was also assigned an overall risk level in the same way based on the maximum contamination level found among the samples collected during the survey.

**Table 1:** Classification of surveyed sites.

Category	Description	Number of sites
private	Residential buildings	250
tourism	Hotels, hostels, beach lodgings	192
elderly	Any facility for old-age people, such as retirement homes and nursing homes	85
other	Shops, offices, industrial buildings, farms	63
health	Health-care facilities, such as hospitals, clinics, and medical offices	63
recreation	Gyms, swimming pools, restaurants, bars	53
school	Schools and kindergartens	24
military	Military buildings	9

### Statistical analysis

Clustered permutation tests accounting for correlation among the samples (with samples clustered by date and location) were used to assess whether contamination levels were different in follow-up surveys compared to non-clinical surveys and in samples collected in hot water circuits compared to samples collected in cold water systems.

Qualitative and quantitative PCR were performed on 390 samples and 42 samples, respectively [27, 33, 34]. PCR was applied only to samples related to clinical cases. Durkalski’s adjustment of the McNemar test for marginal homogeneity accounting for correlated data [35] was used to assess whether qualitative PCR results differed significantly from culture.

The raw data contained 852 distinct descriptions of locations (municipality plus details) with varying degrees of accuracy, most of which were mapped to 739 distinct sites. Population data for each municipality during the period of study were retrieved from the Italian National Institute of Statistics (ISTAT). Further spatial features (e.g., town boundaries and building classification) were retrieved from Friuli Venezia Giulia’s Regional Infrastructure of Environmental and Territorial Data (IRDAT) and from OpenStreetMap. Data processing and analysis was carried out with OpenStreetMap Nominatim, PostGIS 2.4, R 3.5.0 and QGIS 2.18.0.

Several types of spatio-temporal analyses were performed using SaTScan v9.6 (https://www.satscan.org/) and the R rsatscan package v0.3.9200. Environmental surveys were used as “cases” (in the terminology adopted by the software), i.e., as units of observation. An ordinal risk level was associated to each survey by categorizing the maximum contamination found in the samples collected during each survey as previously explained. Accordingly, for all the analyses an ordinal model [36] with risk level as a covariate was used to identify potential clusters with an excess of cases in the high-valued categories of risk. How SaTScan works is best described for purely spatial clustering. In a purely spatial analysis, the software considers, for each location, the collection of all circular areas centered at that location up to a predefined size, and computes, for each area, a statistic based on the likelihood ratio test. The most likely cluster is then defined as the area with the maximum value of the likelihood ratio test statistic. The exact formula of the statistic is quite complex and can be found in [36]. The null hypothesis for the test is that the probability of an observation belonging to a given category is the same inside and outside the scanned area. That is, if *p*_*j*_ is the (unknown) probability that a survey in a certain zone *A* has an associated risk level *j* and *q*_*j*_ is the (unknown) probability that a survey outside *A* has an associated risk level *j*, then the null hypothesis is *H*_0_ : *p*_1_ = *q*_1_, …, *p*_*k*_ = *q*_*k*_, where 1, …, *k* are the *k* risk levels. The alternative hypothesis is *H*_*a*_ : *p*_1_/*q*_1_ ≤ ⋯ ≤ *p*_*k*_/*q*_*k*_, with at least one inequality being strict. This ensures that the detected clusters are associated with a higher risk compared to the surrounding area. As a further refinement, the observed rates are not required to be strictly in increasing order for all categories, because different categories may be aggregated. For example, a cluster may be detected with a rate of high-risk surveys that is unusually high compared to low-risk and medium-risk surveys *combined*, even if the rate of medium-risk surveys may not be as high as the rate of low-risk surveys. Another important feature of this method is that it takes into account the actual spatial distribution of the observations and not the total numbers observed, which was very important in the considered setting, as the spatial distribution of surveys was extremely skewed. For purely temporal clustering, the search strategy is similar, but the scanning zones are temporal intervals of variable duration rather than spatial circles; for spatio-temporal analyses, the scanning zones can be thought of as cylinders whose base is a circular area and whose height is a temporal interval.

Retrospective purely temporal, purely spatial, spatio-temporal and seasonal analyses were performed using the ordinal model described above. Non-clinical and follow-up surveys were analyzed separately, and separate analyses were also run on subsets of the data corresponding to specific types of surveys (e.g., surveys performed in health care facilities, surveys performed in touristic places, and so on). Follow-up surveys in residential buildings were typically performed once for each address; in the few cases where a second survey was conducted at the same location (usually within 30 days since the first survey), only the first result was kept for the purpose of clustering. Purely spatial analysis was used to detect zones or sites where an excess of surveys with higher risk levels was repeatedly found. The purely temporal and seasonal ordinal statistics were also applied to evaluate peaks in the spreading of the bacteria and the potential recurrence of outbreaks. For the purely temporal model, clusters of size between one day and approximately two and a half years (15% of the study period) were considered. In both the purely temporal and seasonal models, the time aggregation length [37] was set to one day. In spatio-temporal analyses, spatially non-overlapping clusters with a temporal range of one month to approximately two and a half years including at most 20% of the surveys were searched.

Given the relatively small size of the region, it was useful to set the maximum radius of a cluster to 5 km; by running simulations with different sets of parameters, it was empirically determined that limiting the spatial extension of the clusters to 1%–5% of the total surveyed region was crucial to detect informative clusters (comparatively, restricting the clusters along the temporal dimension was not as important); without such constraint SaTScan tended to aggregate several small clusters into one large cluster (compared to the overall size of the region), obscuring the real “hot spots”. For spatio-temporal clustering, the time aggregation length was set to three months, mainly for computational reasons: such aggregation significantly reduced the required computation time without overly affecting the results. Finally, two prospective spatio-temporal analyses were simulated, one during the period 2006–2009 and the other during 2015–2017, in which periodic searches for clusters ending at the current date were repeated every two weeks. Prospective analyses only find “alive” clusters, i.e., clusters that last until the current time, so they are particularly useful as predictive tools for early detection of areas with an unusual increase in the risk levels. The time aggregation length in this case was set equal to the interval between two analyses, i.e., two weeks [37], and the results were compared to those obtained by the retrospective analyses. In all cases, statistical significance was assessed using SaTScan’s default option combining three different methods based on Monte Carlo simulations (*N* = 999) and the threshold for statistically significant *p*-values was set to 0.05.

## Results

### Non-clinical surveys

**Fig 1:**
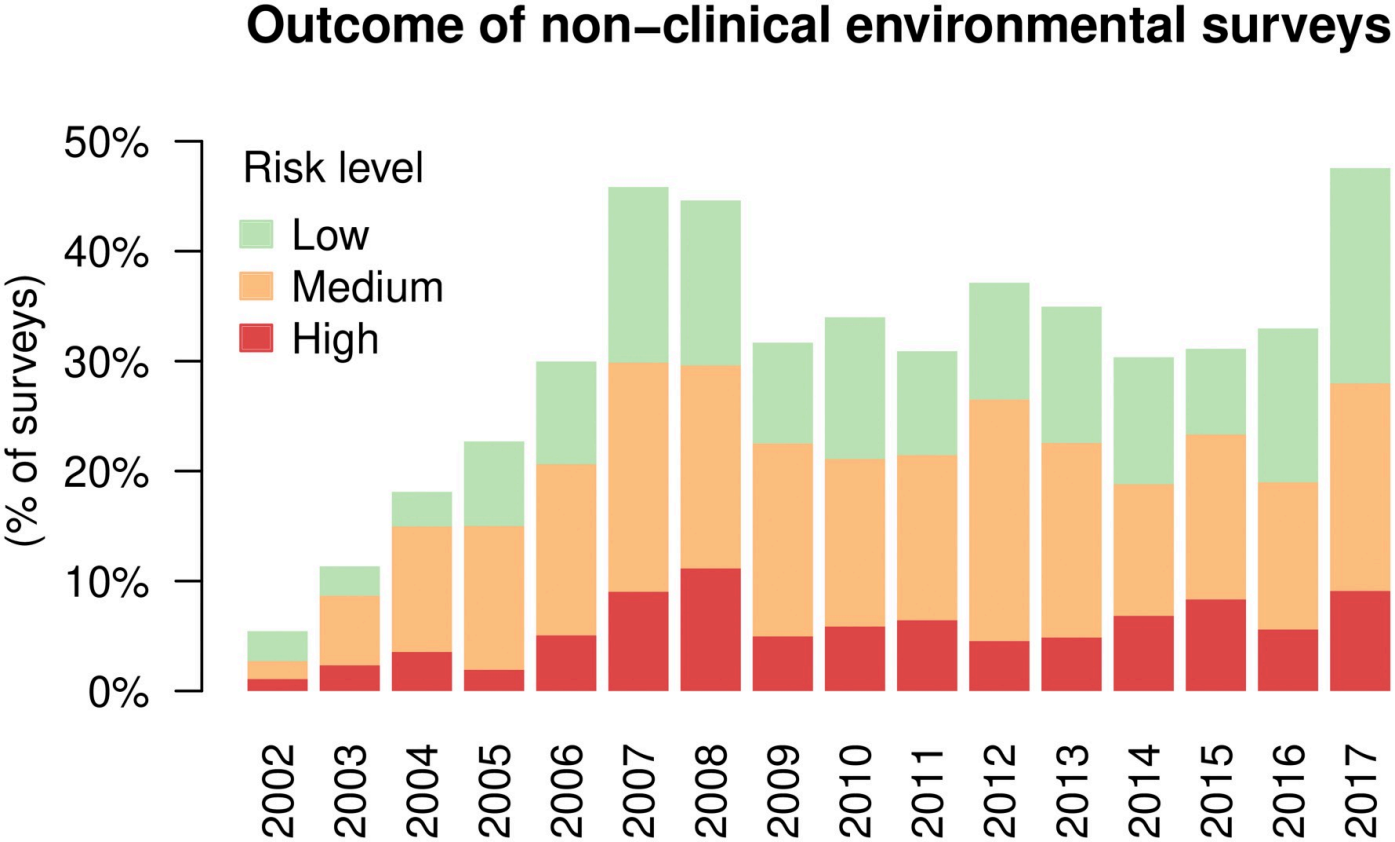
Outcome of non-clinical environmental surveys. Percentage of low (100 ≤ CFUl^−1^ ≤ 1,000), medium (1,000 ≤ CFUl^−1^ ≤ 10,000) and high (>10,000 CFUl^−1^) risk surveys for each year (see also Table 2).

During the period of study, 3,648 non-clinical surveys were routinely performed at 471 sites for a total of 18,104 harvested samples. *Legionella* was found in 3,585 (19.8%) samples from 180 sites (38.2% of surveyed sites). Among the quantified samples above the limit of detection (3,581 samples, 4 samples were recorded only as “positive”), the percentages of contamination levels ≤1,000, 1,001–10,000, and ≥10,000 CFUl^−1^ were 48.6% (1739 samples), 41.4% (1481 samples) and 10.1% (361 samples), respectively. An average of 8 surveys were conducted at each location during the period of study, although the number of surveys per location was greatly skewed (median: 2, 3^rd^ quartile: 9; see also S2 Fig); 45% of the sites were surveyed only once. An average of 40 samples were collected at each site during the whole period of study (median: 8, 3^rd^ quartile: 34), and an average of 5 samples were harvested in each survey (median: 4). The outcome of each survey during the whole period of study, highlighting the risk levels, is summarized in Fig 1 (see also S1 Table). The nosocomial sector was more affected by the presence of *Legionella* compared to the other categories (see Table 2 and S2 Table).

**Table 2:** Non-clinical surveys by category of settings. Each survey with at least one positive sample was assigned a “positive” outcome and was further classified into low (100 ≤ CFUl^−1^ ≤ 1,000), medium (1,000 ≤ CFUl^−1^ ≤ 10,000) and high (>10,000 CFUl^−1^) risk based on the highest contamination found. Table rows were sorted in decreasing order with respect to the percentage of positive surveys. All surveys in the “private” category were follow-up surveys (hence, not shown here).

Category	N. surveys	Positive		Low risk		Medium risk		High risk	
		N.	Perc.	N.	Perc.	N.	Perc.	N.	Perc.
Health	824	433	52.5%	111	25.6%	221	51.0%	101	23.3%
Military	21	9	42.9%	3	33.3%	3	33.3%	3	33.3%
Elderly	1107	301	27.2%	94	31.2%	158	52.5%	49	16.3%
School	27	7	25.9%	5	71.4%	0	0.0%	2	28.6%
Other	78	18	23.1%	9	50.0%	4	22.2%	5	27.8%
Recreation	351	75	21.4%	38	50.7%	35	46.7%	2	2.7%
Tourism	1240	255	20.6%	105	41.2%	107	42.0%	43	16.9%
Total	3648	1098	30.1%	365	33.2%	528	48.1%	205	18.7%

Typing information was recorded for 3,580 samples. The most frequent serogroup was *Legionella pneumophila* serogroup (*L. pn* sg) 2-15 (62.9%, 2254 samples), followed by *L. pn* sg 1 (35.9%, 1288 samples) and *L. sp*. non-*pneumophila* (1.1%, 38 samples). For 11,141 samples, a qualitative description of the water circuit (cold/hot) was recorded (see Materials and methods). No significant differences between contamination levels in cold and hot water circuits were found (*p* ≥ 0.1), although medium-risk and high-risk samples were found more often in hot water systems (S3 Table). Samples were collected using an instantaneous or quantitative method (see Materials and methods) in equal proportion. The sampling procedure did not appear to be associated with any of the above mentioned variables.

### Hospitals

**Fig 2:**
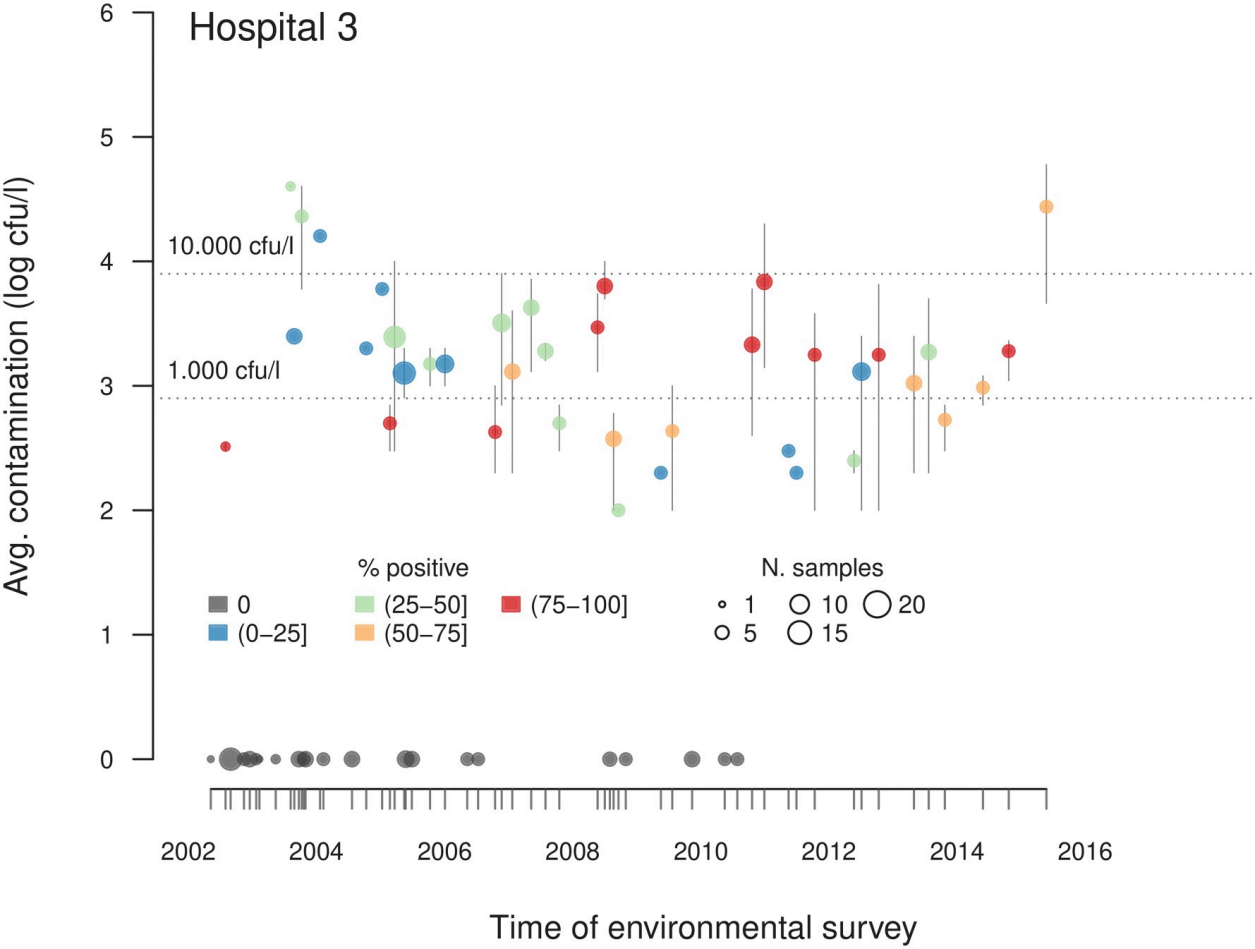
Sample data from environmental surveys conducted in hospitals. Results of surveys from one of the main hospitals of Friuli Venezia Giulia, Italy since 2002 to 2017 are shown. Each data point represents the average positive contamination level of a survey (i.e., it is the average of the contamination levels of the positive samples). The vertical bars indicate the minimum and maximum positive contamination level. The gray dots correspond to surveys in which all the samples resulted negative.

Overall, 824 environmental surveys were performed in 63 health care facilities, and *Legionella* was found at least once in 32 of them (50.8%). Serogroup *L. pn* sg 2-15 accounted for 73.0% of all the samples, and *L. pn* sg 1 accounted for 26.7%. Of the 824 surveys, 479 were conducted in the 9 main hospitals of the region and *Legionella* was found recurrently in all hospitals (Fig 2, Table 3, S4 Table and S3 Fig). In six of the hospitals, contamination was absent or sporadic before 2005–2007, but the pathogen was observed repeatedly afterwards. In two other hospitals, positivity was evenly distributed over the whole period of study. In another hospital, investigations started in 2010, and during the period from 2010–2014, all surveys except one were positive; after 2014, the effects of systematic sanitization were apparent, as an increased number of negative results were observed, although the pathogen was still periodically found, showing that it was not eradicated completely.

**Table 3:** Surveys in the main hospitals. Summary statistics about environmental surveys (top) and collected samples (bottom) from the nine main hospitals of Friuli Venezia Giulia, Italy are shown. Each survey with at least one positive sample was assigned a “positive” outcome and was further classified into low (100 ≤ CFUl^−1^ ≤ 1,000), medium (1,000 ≤ CFUl^−1^ ≤ 10,000) and high (>10,000 CFUl^−1^) risk based on the highest contamination found. Data were sorted by decreasing percentage of positive surveys.

Hospital	Tot.	Positive		Low risk		Medium risk		High risk	
		N.	Perc.	N.	Perc.	N.	Perc.	N.	Perc.
**Surveys**									
Hs-6	52	43	82.7%	6	14.0%	25	58.1%	12	27.9%
Hs-5	33	27	81.8%	1	3.7%	9	33.3%	17	63.0%
Hs-3	99	68	68.7%	19	27.9%	40	58.8%	9	13.2%
Hs-7	42	28	66.7%	7	25.0%	14	50.0%	7	25.0%
Hs-4	59	38	64.4%	12	31.6%	21	55.3%	5	13.2%
Hs-9	48	28	58.3%	10	35.7%	17	60.7%	1	3.6%
Hs-1	55	32	58.2%	8	25.0%	15	46.9%	9	28.1%
Hs-2	62	31	50.0%	5	16.1%	13	41.9%	13	41.9%
Hs-8	31	14	45.2%	7	50.0%	6	42.9%	1	7.1%
Total	481	309	64.2%	75	24.3%	160	51.8%	74	24.0%
**Samples**									
Hs-6	700	319	45.6%	172	53.9%	126	39.5%	21	6.6%
Hs-5	364	198	54.4%	37	18.7%	117	59.1%	44	22.2%
Hs-3	1148	314	27.4%	185	58.9%	117	37.3%	12	3.8%
Hs-7	599	161	26.9%	78	48.4%	64	39.8%	19	11.8%
Hs-4	297	107	36.0%	53	49.5%	48	44.9%	6	5.6%
Hs-9	195	58	29.7%	35	60.3%	22	37.9%	1	1.7%
Hs-1	411	118	28.7%	53	44.9%	55	46.6%	10	8.5%
Hs-2	487	120	24.6%	35	29.2%	57	47.5%	28	23.3%
Hs-8	298	43	14.4%	26	60.5%	14	32.6%	3	7.0%
Total	4499	1438	64.2%	674	46.9%	620	43.1%	144	10.0%

From the written annotations for approximately 1,815 of the samples collected in health care facilities, it was possible to extract information about the unit where each sample was collected. *Legionella* was transversally present within all the wards, but with large differences across wards (see Table 4 and S5 Table).

**Table 4:** Outcome of environmental surveys by hospital ward. Each survey with at least one positive sample was assigned a “positive” outcome and was further classified into low (100 ≤ CFUl^−1^ ≤ 1,000), medium (1,000 ≤ CFUl^−1^ ≤ 10,000) and high (>10,000 CFUl^−1^) risk based on the highest contamination found. Data were sorted by decreasing percentage of positive surveys. Only units in which at least 30 samples were collected during the period of study were considered.

Unit	N. wards	N. surveys	Positive		Low		Medium		High	
			N.	Perc.	N.	Perc.	N.	Perc.	N.	Perc.
Hematology	2	24	18	75.0%	8	44.4%	10	55.6%	0	0.0%
Neonatology	5	52	38	73.1%	16	42.1%	18	47.4%	4	10.5%
Oncology	10	51	28	54.9%	8	28.6%	15	53.6%	5	17.9%
Obstetrics	10	71	38	53.5%	4	10.5%	23	60.5%	11	28.9%
Orthopedics	14	98	48	49.0%	16	33.3%	27	56.2%	5	10.4%
Neurology	5	16	7	43.8%	2	28.6%	2	28.6%	3	42.9%
Pediatrics	12	89	38	42.7%	15	39.5%	18	47.4%	5	13.2%
Surgery	17	165	68	41.2%	25	36.8%	31	45.6%	12	17.6%
Dialysis	11	44	18	40.9%	2	11.1%	8	44.4%	8	44.4%
Spinal unit	3	34	12	35.3%	7	58.3%	5	41.7%	0	0.0%
Cardiology	8	20	7	35.0%	3	42.9%	4	57.1%	0	0.0%
Intensive care	8	37	9	24.3%	2	22.2%	7	77.8%	0	0.0%
Day Hospital	6	15	2	13.3%	0	0.0%	2	100.0%	0	0.0%
Total	111	716	331	46.2%	108	32.6%	170	51.4%	53	16.0%

### Elderly, tourism, and recreation

Trends for retirement homes (S4 Fig), hotels (S5 Fig) and swimming pool environments (S6 Fig) was more varied compared to hospitals, but the data confirmed that *Legionella* was able to persist in complex water distribution systems [27].

During the period of study, 80 facilities for elderly people were surveyed 1,107 times, during which 5,289 samples were collected, of which 839 were positive. *Legionella* was found at least once in 47 sites (57.8%). Three types of trends could be recognized in nursing and retirement homes (RH1 through RH12): (a) the presence of *Legionella*, except perhaps at the beginning of the period of study, was more or less stable or recurrent (RH1, RH5, RH11 in S4 Fig), similar to what was found in hospitals; (b) disinfection procedures were effective in eradicating the bacteria for a few years, but the pathogen was found again in medium/high levels afterwards (RH2, RH3, RH4, RH7, RH8); (c) contamination, possibly after sanitization, remained sporadic and in low/medium levels, or even totally absent (RH6, RH9, RH10, RH12). The most frequent serogroup in the elderly category was *L. pn* sg 2-15 (49.9%, 419 samples), followed by *L. pn* sg 1 (48.0%, 403 samples) and *L. sp*. non-pneumophila (1.9%, 16 samples). The analysis of hotels and swimming pools showed similar trends. Overall, 185 different touristic buildings were surveyed 1,240 times, during which 4,900 samples were collected, and *Legionella* was found at least once in 65 buildings (35.1%). The prevalent serogroup in touristic facilities was *L. pn* sg 2-15 (61.5%, 428 samples), followed by *L. pn* sg 1 (36.8%, 256 samples) and *L. sp*. non-pneumophila (1.7%, 12 samples). Of the 51 sites classified as *recreational* (including gyms, swimming pools, restaurants, and bars), *Legionella* was found in 10 (18.9%), 9 of which were swimming pool environments (50.0% all of surveyed swimming pools). In swimming pool environments, the most frequent serogroup was *L. pn* sg 1 (82.4%, 103 samples), followed by *L. pn* sg 2-15 (12.8%, 16 samples) and *L. sp*. non-pneumophila (4.8%, 6 samples). Swimming pool environments are notoriously subject to constant and programmed disinfection cycles, but at the same time, they have a high number and multiple types of hot water dispensers, wet surfaces and heating, ventilation and air-conditioning systems (HVAC), which favor the diffusion of *Legionella*. In one case, *Legionella* reappeared after almost a decade (2005–2015) during which all surveys except one had given negative results.

### Spatio-temporal analysis

#### Spatio-temporal clustering

**Fig 3:**
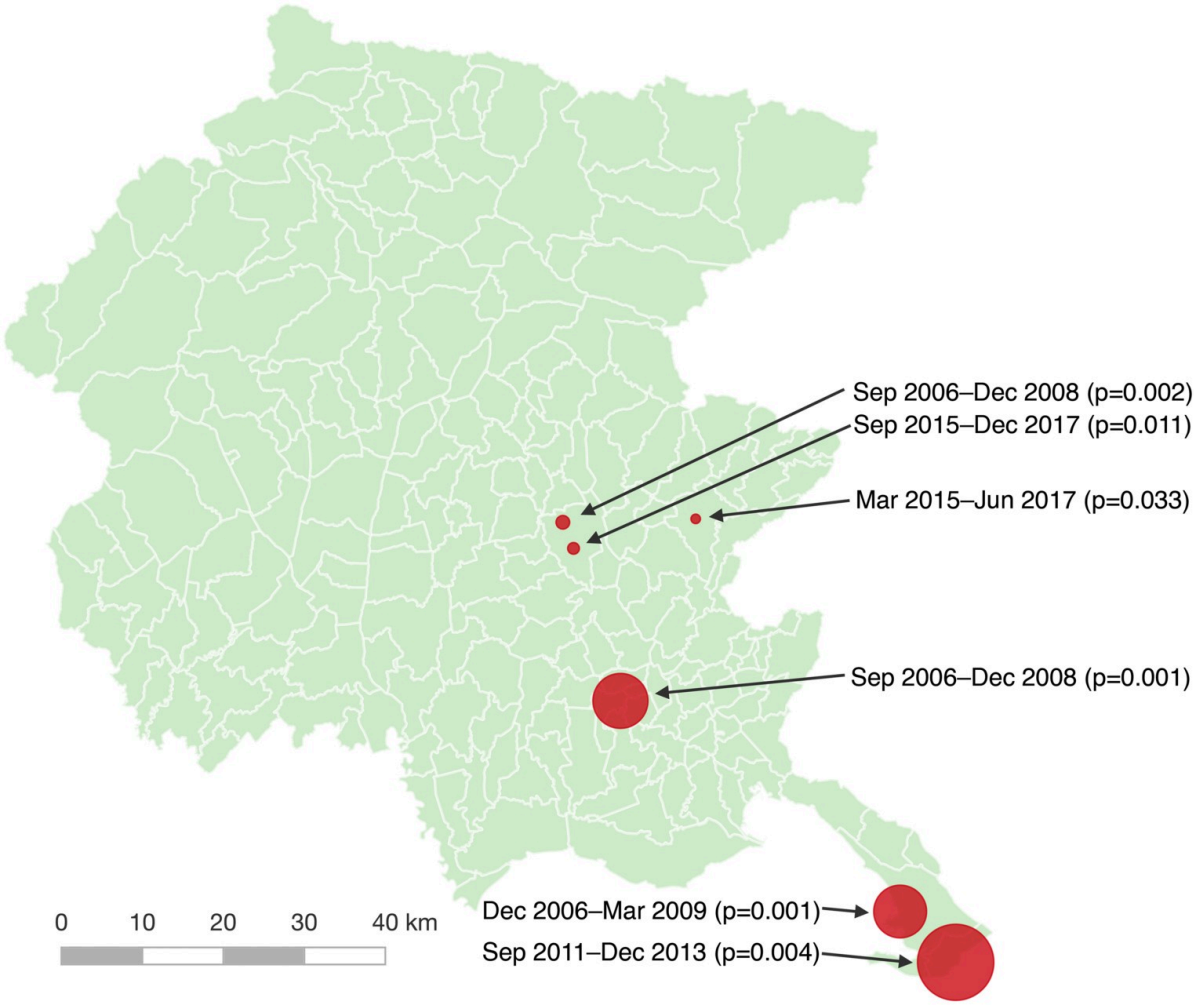
Spatio-temporal clusters of non-clinical environmental surveys. The figure shows the time and location of the 6 statistically significant clusters (*p* ≤ 0.05) of non-clinical environmental surveys found using an ordinal model with categorized contamination levels of surveys as a covariate [36, 37].

A retrospective spatio-temporal analysis based on the ordinal model and using the time and location of all the non-clinical surveys as units of observation identified 16 clusters, of which 6 reached statistical significance (see S6 Table). Fig 3 illustrates the geographical location of each cluster. Clusters tended to form around health care structures. Although this was not surprising given the regular presence of *Legionella* in hospitals, the results were still informative because (a) they delimited the clusters temporally and (b) they highlighted interesting relationships with the surrounding area. For instance, Cluster ST5 included three nearby hotels in which medium to high contamination levels were repeatedly found between September 2015 and December 2017. The spatial area covered by Cluster ST5, which was only 1.6 km^2^, included 14 sites surveyed during the whole period of study, 6 of which were private residential buildings that were subject to environmental inspections as a consequence of reported cases of legionellosis in 2002, 2003–2004, 2007, 2008, 2011, and 2012. Note that data from follow-up surveys were not used for this analysis.

#### Purely spatial clustering

**Fig 4:**
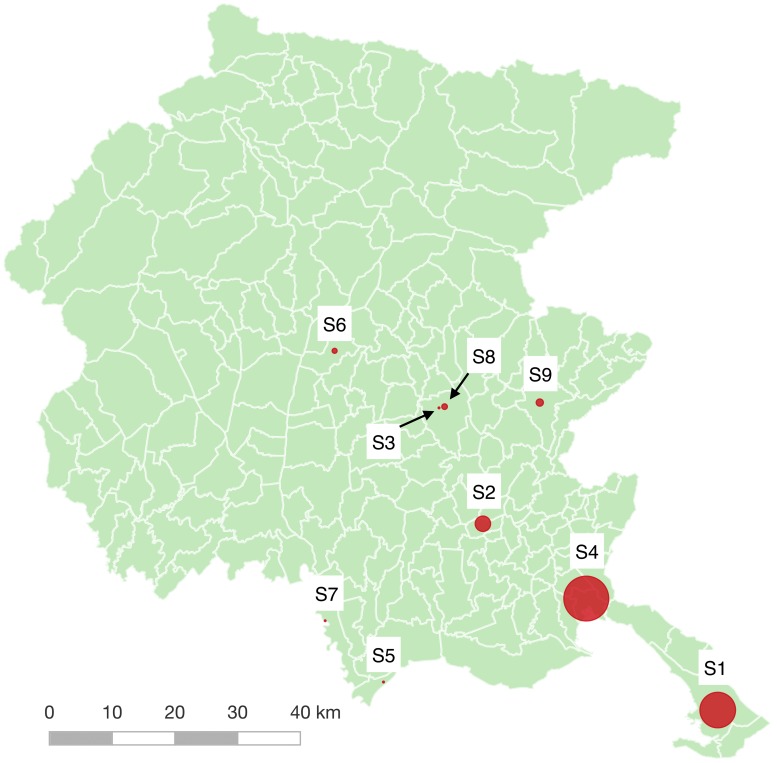
Purely spatial clusters of non-clinical environmental surveys. The figure shows the location of the 9 statistically significant spatial clusters (*p* ≤ 0.05) of non-clinical environmental surveys found using an ordinal model with categorized contamination levels of surveys as a covariate [36, 37].

A retrospective purely spatial analysis found 21 clusters, 9 of which were statistically significant (see Fig 4 and S7 Table). Since this analysis did not use the temporal dimension, each cluster contained more observations, and *p*-values were much lower than in the spatio-temporal case. In fact, some clusters (Clusters SP5, SP6, SP7, and SP9) were also found by the spatio-temporal analysis, although in that case they were not statistically significant. Cluster SP8 (0.6 km^2^) was particularly interesting because it was identified using only three locations: a retirement home and a hotel with two buildings two hundred meters apart from each other. The remaining four sites inside Cluster SP8 were private residential buildings in which follow-up surveys (not used for this analysis) were conducted in 2008, 2012, 2013–2014 and 2017.

#### Purely temporal clustering

A retrospective purely temporal analysis was performed on the whole data set and for surveys in each category (S8 Table). This analysis confirmed that the period between the second half of 2006 and the beginning of 2009 was a peak period for environmental contamination in the region.

#### Seasonality

**Fig 5:**
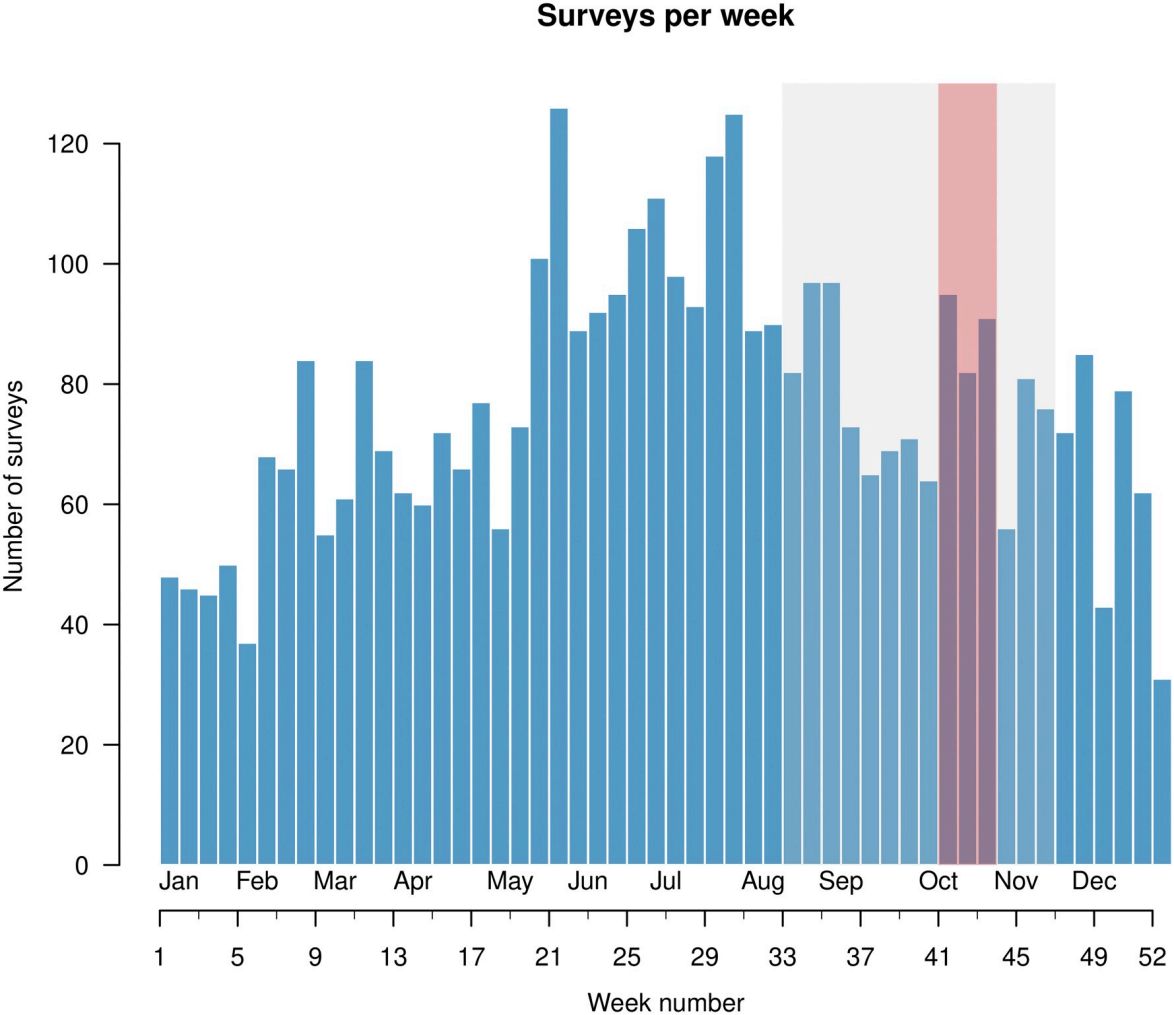
Seasonal clusters. The gray-shaded area covers the statistically significant seasonal clusters (*p* ≤ 0.05) found by applying an ordinal model with categorized contamination levels as a covariate [36, 37] to the subsets of the whole set of non clinical-surveys partitioned by type of building (see Table 1), while the red shaded area is the most likely seasonal cluster associated with all non-clinical surveys.

A temporal analysis based on the seasonal statistic highlighted the end of the summer (August–November) as the period of highest risk (Fig 5 and S9 Table). Note that the period in which the weekly number of surveys had a peak was May through July.

#### Prospective spatio-temporal analysis

The retrospective spatio-temporal and purely temporal analyses confirmed that years 2006–2009 and 2015–2017 were periods with significantly increased contamination, at least in certain areas. As the next step, a periodic prospective analysis was performed to assess how early such clusters could be found (provided that they could be found at all). Simulations were run using data accumulated every two weeks starting at the beginning of 2006 up to the beginning of 2009, searching for spatio-temporal clusters extending to the current date, and the first appearance of each statistically significant cluster, i.e., the date when a statistically significant cluster was first reported by the software, was recorded. Then, the same was done for the period 2015–2017.

**Fig 6:**
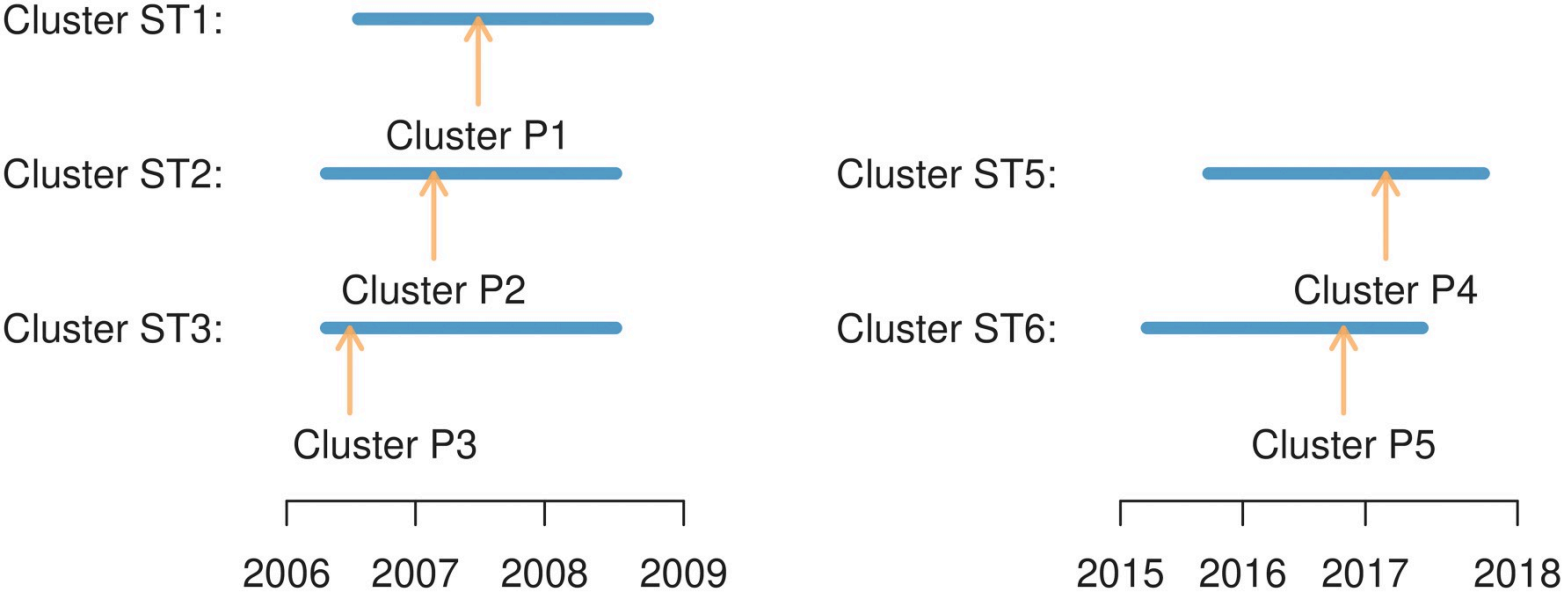
Prospective cluster detection. Each interval represents the temporal span of a retrospective spatio-temporal cluster (see Fig 3 and S6 Table). The arrows indicate the time when the same cluster was found by a prospective simulation (see S10 Table). In all cases, the clusters were reported a few weeks to several months before their end.

All the clusters found by the retrospective analysis were also found prospectively several months before their end (see Fig 6). The relevant information about the earliest statistically significant clusters entirely covering the spatial part of the corresponding retrospective reference cluster is summarized in S10 Table. In most cases, clusters largely overlapping or properly contained inside the corresponding retrospective clusters were found much earlier than the dates shown in S10 Table. For instance, a cluster spatially included in Cluster P1 (which starts on December 2006) and overlapping Cluster ST1 was reported as early as the end of July 2006 based on 6 surveys (*p* = 0.049). For comparison, the reference retrospective Cluster ST1 starts on December 2006. Clusters overlapping ST1 were found on every analysis after July 2006, and since November 2007, the reported clusters were essentially the same size as Cluster ST1. Similarly, Cluster ST2 was covered by Cluster P2 in July 2007, approximately 10 months after the retrospective starting date, but an overlapping cluster had been reported 3 months before based on 5 surveys. Cluster P3, spatially covering Cluster ST3, was found quite early, in November 2006, based on 5 surveys (Cluster ST3 starts in September 2006). Cluster P4, covering Cluster ST5, was reported as statistically significant at the beginning of March 2017 and Cluster P5, covering Cluster ST6, as of October 2016. Both clusters, however, had been reported (with *p*-values greater than 0.05) since May 2015.

### Environmental surveys related to cases of legionellosis

During the period of study, 335 environmental surveys were conducted at 282 different sites as a response to reported cases of legionellosis, and 2,215 samples were collected during such surveys; in 84 surveys (25.1%) from 69 sites at least one sample was positive. The maximum contamination levels ≤1,000, 1,001–10,000, and ≥10,000 CFUl^−1^ reported for such surveys were 35.7% (30 surveys), 41.7% (35 surveys) and 22.6% (19 surveys), respectively. Among all the samples, 355 (16.0%) were positive. Among the positive samples, the percentages of contamination levels ≤1,000, 1,001–10,000, and ≥10,000 CFUl^−1^ were 48.5% (172 samples), 38.9% (138 samples) and 12.7 (45 samples), respectively. The most frequent serogroup was *L. pn* sg 1, with 249 (70.7%) samples, followed by *L. pn*. sg 2-15 with 94 (26.7%) samples and *L. sp*. non-pneumophila with 9 samples (2.6%). Most of the surveys (88.1%, 295 surveys) were performed in private residential buildings.

**Fig 7:**
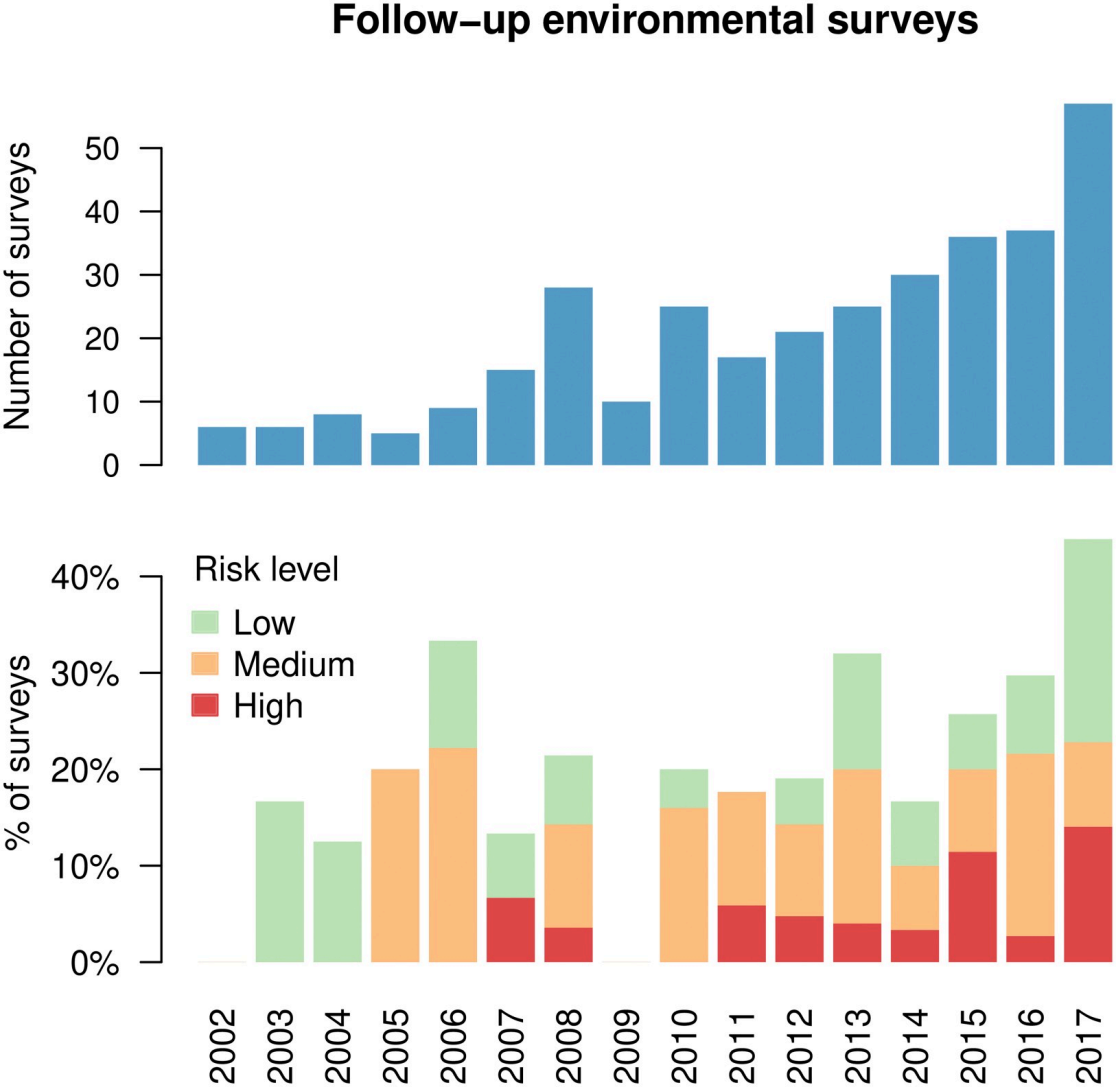
Statistics on follow-up surveys. Each survey was classified as no risk (<100 CFUl^−1^), low risk (100 ≤ CFUl^−1^ ≤ 1,000), medium risk (1,000 ≤ CFUl^−1^ ≤ 10,000) or high risk (>10,000 CFUl^−1^) based on the maximum observed contamination level. In 2002 (6 surveys) and 2009 (10 surveys) no positive samples were found during follow-up surveys.

Although the trend was towards an increasing number of cases of legionellosis being recorded (see Fig 7), with a trend towards wider geographical extent (see S7 Fig), using the number of environmental surveys as an estimate of the number of clinical cases is likely not accurate, as effective compliance with Italian regulations mandating the registration of each clinical case is a confounding factor.

No significant difference was found between the distributions of risk levels in follow-up and non-clinical surveys (*p* ≥ 0.08) (Table 5), although extremely high levels of contamination (above ≥10^5^ CFUl^−1^) were never found in follow-up surveys.

**Table 5:** Comparison of risk levels in non-clinical and follow-up environmental surveys. Each survey was classified as no risk (<100 CFUl^−1^), low risk (100 ≤ CFUl^−1^ ≤ 1,000), medium risk (1,000 ≤ CFUl^−1^ ≤ 10,000) or high risk (>10,000 CFUl^−1^) based on the maximum observed contamination level.

	None	Low	Medium	High
**Non-clinical surveys**	80.2%	9.6%	8.2%	2%
**Follow-up surveys**	83.9%	7.8%	6.3%	2%

Qualitative PCR was applied to 390 samples harvested in 92 follow-up surveys (PCR was not performed on non-clinical samples). For 61 samples PCR was positive, but culture was negative, while PCR was negative and culture positive only in 2 cases, which was attributed to analytical errors. So, PCR analyses found positive results more often (Durkalski’s test: *χ*^2^ = 25.6, *p* < 10^−6^), probably revealing cells in a viable but not culturable (*VBNC*) state [8]. Besides, for 30 samples collected at 11 different sites, both culture and quantitative PCR were performed. Although the two variables appeared to be associated, there was not enough data to assess the nature of the association in a statistically significant way. In particular, for contamination levels above 10,000 CFUl^−1^, no quantitative PCR was available.

A retrospective space-time analysis using the ordinal model on follow-up surveys identified 7 clusters. Given the limited number of observations, none of them reached statistical significance (*p* ≥ 0.09). Most clusters were in the period between 2015 and 2017 (only one cluster fell between 2012 and 2014), consistent with the fact that a high percentage of cases (39%) were reported after 2015. A purely spatial analysis found 5 clusters (*p* ≥ 0.052), the most significant one coinciding the most significant cluster found by the spatio-temporal analysis.

## Discussion

We have analyzed a large data set of historical data about environmental surveys performed in Friuli Venezia Giulia, Italy, over a time span of approximately 16 years. The region has not witnessed any outbreaks during this period, and the adopted policies have helped keep the diffusion of Legionnaires’ disease under control. According to the notifications received by ARPA-FVG, however, the incidence of hydrodiffused diseases has shown an increasing trend. In particular, the number of reported cases of legionellosis has steadily increased since 2002 (<6 cases) to 2017 (32 cases). While increasing awareness and compliance with mandatory regulations likely play a role, to explain such a trend it must be acknowledged that the spreading of the disease may be a growing phenomenon.

A feature that distinguishes this study from most other studies about *Legionella* is that only environmental data were used; i.e., no clinical information was available (except that it was known whether an environmental survey was conducted after a reported case of legionellosis). The goal was to assess to what degree such information would allow us to locate areas with an “unusual” presence of *Legionella* and, especially, whether we would be able to detect an onset of the spreading of the pathogen in the environment. Given the highly correlated nature of the data, given the fact that spatial autocorrelation was not adjusted for (in fact, we wanted to test whether clusters existed *due* to such autocorrelation) and given the accepted classification of contamination levels into few categories, the most convenient tool for such analysis was the ordinal spatio-temporal model implemented by SaTScan [36]. The availability of thousands of space-time geolocated environmental surveys allowed us to tune the parameters of the model so that a reliable statistical approach could be built. This, on the one hand, confirmed what it was already possible to determine from a descriptive analysis of the data (for instance, the periods of highest contamination), and, on the other hand, allowed us to gain a deeper insight into the data. Clusters determined using only information about environmental inspections may highlight zones, or even sites, that require special attention by the local health authorities, either because they have historically been subject to recurrent episodes or persistent contamination, or, prospectively, because they emerge as areas with increasing colonization. A question that naturally arises is whether clusters of environmental *Legionella* are somehow associated with clinical cases. Although we pointed out at some evidence in this sense (see Results) and although we found that areas inside or in the proximity of each cluster always contained the location of at least one follow-up survey, the data at our disposal did not allow us to answer such question conclusively, the main reason being that follow-up surveys provide only an approximation of the real source of infection. Also, as we suggest below, it may be that even areas with low or medium contamination levels may be dangerous: a model aimed at detecting high environmental contamination would not report such areas.

The data also strongly hint at a seasonal behavior of the pathogen, with a peak in early autumn, confirming what has been reported in the literature [38]. Finally, the proposed method appears to be very sensitive; in some cases, less than five environmental surveys within a limited spatial or spatio-temporal frame were sufficient to report a cluster. This is especially important in prospective analyses, where early detection of potentially anomalous situations can greatly help in terms of prevention and control. Furthermore, the simulated prospective analyses allowed us to detect more clusters than retrospective analyses, and in general, they provided a more dynamic view of clusters’ development. For instance, a statistically significant cluster (*p* = 0.018) in the same area as Cluster ST6 (March 2015 to June 2017) was also found prospectively in the period from August 2006 to September 2007, suggesting a recurrence of environmental contamination in that area that was not previously known. The retrospective and prospective analyses that we have done suggest that the proposed model has the potential to identify and locate epidemic clusters in advance. This tool might support public health workers to guide interventions and reduce the costs of systematic and nontargeted operations.

An indicator that has been used by reference regional centers in Italy as a flag for a potential outbreak is based on targeting sites where at least two cases of legionellosis are reported within a two-year period. The method we propose generalizes such criterion by (a) considering variable-length temporal windows, (b) taking into account wider spatial areas including several buildings, neighborhoods or districts, and (c) incorporating environmental data rather than or in addition to clinical data. While we do not suggest that currently used indicators should be changed, we believe that complementary tests could help health care operators provide better responses to environmental changes. Spatio-temporal clustering might prove to be an effective component of a surveillance system for the timely detection of unusual spatially and temporally localized peaks in incidence. Simulations suggest that outbreaks might be anticipated or promptly detected using the proposed method paired with careful monitoring of the environment. An important consideration in this sense is that the proposed method should not be used for planning because of the risk of a feedback loop that would bias the cluster analyses; instead, a monitoring strategy developed on independent criteria should be adopted and the collected data subsequently analyzed using SaTScan.

Another interesting finding is that the distribution of contamination levels in follow-up and non-clinical surveys did not differ in significant ways, whereas it was expected to find high contamination levels more often in follow-up surveys. Instead, 87.4% of the positive samples collected in follow-up surveys were below the 10,000 CFUl^−1^ threshold (82.3% if we consider only *L. pn* sg 1 samples). Besides, of the 69 sites where the pathogen was found, only 23 (33.3%) had contamination levels above 10,000 CFUl^−1^. Although the available data did not allow us to establish with certainty that the surveyed sites were the source of the disease in each case, such numbers are striking as being quite low, indicating that the disease may be contracted from water contaminated at levels well below the 10,000 CFUl^−1^ threshold.

If most human infections are caused by *L. pn* sg 1 [39], which is supported by our analysis of follow-up surveys, non-clinical investigations have instead identified a greater diffusion of *L. pn* sg 2-15 in the environment, especially in hospitals (>74%). For future work, it might be worth investigating the effects of taking into account the serogroup in the assessment of the risk associated with clustered surveys.

Trends for health care facilities, and hospitals in particular, appear to be different from the other categories, showing a persistent presence of the bacteria even after programmed interventions, indicating that it is very difficult to neutralize *Legionella* in large, and possibly old, building complexes. The current state of the knowledge of the diffusion of *Legionella* in the environment still limits the effectiveness of the actions undertaken [16]. Unfortunately, information regarding variables concerning the management and control of the water system facilities, as well as information about the age and state of the buildings, were not available to us. Regarding facilities for elderly people (retirement homes, rest homes, and nursing homes), it is important to reduce contamination sources since the incidence of Legionnaires’ disease increases with age [26]. It has been suggested that in those facilities the aerosol generated while showering is the main route of exposure to *Legionella* [40]. Additionally, *Legionella* has been found primarily in water samples rather than in biofilms [41]. In swimming pool environments, the risk of legionellosis is associated with the proliferation of *Legionella* in spas, whirlpools, associated equipment and HVAC systems, although showers may present a greater risk of legionellosis than pool water [42]. Environmental factors contributing to the diffusion of *Legionella* in such environments include inadequate water treatment (residual disinfectant concentration below recommended levels), water temperature above 40°C, and inadequate structure maintenance. Inadequacy or absence of the treatment system was observed for cases or outbreaks verified in private swimming pool environments not subjected to an additional disinfection system [42].

Among the reasons why all the above mentioned facilities are affected by *Legionella* contamination, low use of hot water or the presence of blocked branches of plumbing that create stagnation of the fluid may be a common factor. Under these conditions, a continuous water chlorination system may not be effective in controlling *Legionella*. It has been shown that the insertion of timed flow taps (TFTs) to increase the flow of hot water recirculation together with the same level of chlorination drastically reduces the presence of *Legionella* [43].

Currently, the improvement of epidemiological studies is mainly concerned with molecular investigation techniques extended to the study and comparison of whole genomes (WGS). A specific potential reservoir may apply a selective pressure on *Legionella* strains, causing different genetic evolutions, which can be used to build filogenetic trees to better understand the processes of diffusion and the phenotipic correlations among the strains. At the same time, such strains are good candidate tools for predictive analysis [44]. A future research trend will involve combining environmental surveillance of *Legionella* with genetic techniques.

## Supporting information

S1 Fig Location of Friuli Venezia Giulia, Italy. Friuli Venezia Giulia, the northeastern-most region of Italy, facing the Adriatic Sea and bordering Austria and Slovenia, has a population of approximately 1,200,000 inhabitants, a high quality of life and a touristic vocation.
(TIF)

S2 Fig Map of surveyed sites and population density. Each dot on the map corresponds to one of the 739 geolocalized sites where at least one survey was conducted during the period of study. The gradient represents the population density as of 2016 for each municipality in the region. The two main cities of Trieste and Udine are the most densely populated areas, while all of the northern part of Friuli Venezia Giulia is mountainous and sparsely populated. Consequently, the spatial distribution of surveys is highly nonuniform.
(TIF)

S3 Fig Surveys in the main hospitals. Each graph shows the data for the samples collected in one of the main hospitals of Friuli Venezia Giulia, Italy (the graph for Hospital 3 in the main article). Each data point represents the average positive contamination level of a survey (i.e., it is the average of the contamination levels of the positive samples of the survey), it is colored according to the percentage of positive samples of the survey, and its size is proportional to the total number of samples harvested during the survey (see the legend in the top-left figure). The vertical bars around each dot indicate the minimum and maximum positive contamination level found during each survey. The gray dots correspond to surveys in which all the samples resulted negative.
(TIF)

S4 Fig Surveys in retirement homes. For interpreting the graphs, see Fig 2.
(TIF)

S5 Fig Surveys in hotels. For interpreting the graphs, see Fig 2.
(TIF)

S6 Fig Surveys in swimming pool environments. For interpreting the graphs, see Fig 2.
(TIF)

S7 Fig Spatio-temporal distribution of legionellosis. The figure shows the location of follow-up environmental surveys performed in Friuli Venezia Giulia after notifications of cases of legionellosis since 2002 to 2017.
(TIF)

S1 Table Yearly survey statistics. From left to right, the table shows, for each year, the overall number and percentage of positive surveys, and the number and percentage of low (100 ≤ CFUl^−1^ ≤ 1,000), medium (1,000 ≤ CFUl^−1^ ≤ 10,000) and high (>10,000 CFUl^−1^) risk surveys, respectively. A survey was considered “positive” when at least one sample associated to the survey tested positive. The risk level of each survey was determined based on the highest contamination level among the samples in the survey.
(PDF)

S2 Table Samples by category of settings. For each category, the total number of samples collected during the period of study and the number and percentage of positive samples are reported. The last three pairs of columns describe the distribution of positive samples across the corresponding risk levels: low (100 ≤ CFUl^−1^ ≤ 1,000), medium (1,000 ≤ CFUl^−1^ ≤ 10,000) and high (>10,000 CFUl^−1^) risk. Table rows are sorted in decreasing order with respect to the percentage of positive samples.
(PDF)

S3 Table Hot vs cold water circuits. Distribution of contamination levels for samples collected in cold water systems (or systems without hot water recirculation) and hot water circuits, respectively, across four risk levels: no risk (<100 CFUl^−1^), low risk (100 ≤ CFUl^−1^ ≤ 1,000), medium risk (1,000 ≤ CFUl^−1^ ≤ 10,000) and high risk (>10,000 CFUl^−1^).
(PDF)

S4 Table Temporal trends in the main hospitals. The table reports summary statistics about environmental surveys (top) and collected samples (bottom) from the main hospitals of Friuli Venezia Giulia. From left to right, the table shows the year, the number of surveyed hospitals, the overall number of conducted surveys (top) or overall number of collected samples (bottom) in each year, the number and percentage of positive surveys/samples, and the number and percentage of low (100 ≤ CFUl^−1^ ≤ 1,000), medium (1,000 ≤ CFUl^−1^ ≤ 10,000) and high (>10,000 CFUl^−1^) risk surveys/samples, respectively. A survey was considered “positive” when at least one sample associated to the survey tested positive. The risk level of each survey was determined based on the highest contamination level among the samples in the survey.
(PDF)

S5 Table Samples by hospital unit. From left to right, the table shows the type of each unit, the number of distinct surveyed wards of each type, the overall number of collected samples for each unit type, the number and percentage of positive samples, and the number and percentage of low (100 ≤ CFUl^−1^ ≤ 1,000), medium (1,000 ≤ CFUl^−1^ ≤ 10,000) and high (>10,000 CFUl^−1^) risk samples, respectively. Data are sorted by decreasing percentage of positive samples. Only units in which at least 30 samples were collected during the period of study are shown.
(PDF)

S6 Table Spatio-temporal clusters of surveys. From left to right, the table shows, for each cluster that reached statistical significance (*p* ≤ 0.05), its identifier, its temporal extension, its area (in km^2^), the overall number of sites falling inside the cluster’s area (indepedent of when they were surveyed), the number of environmental surveys conducted inside the area delimited by the cluster within the corresponding time frame, the ratio between observed and expected number of surveys for each risk level, the relative risk for each risk level (which represents how much more common surveys with a given risk level are compared to the baseline), the log-likelihood ratio and the *p*-value associated with each cluster. A risk level was associated to each survey based on the highest contamination level among the samples collected in each survey, as follows: no risk (<100 CFUl^−1^), low risk (100 ≤ CFUl^−1^ ≤ 1,000), medium risk (1,000 ≤ CFUl^−1^ ≤ 10,000) and high risk (>10,000 CFUl^−1^). Data are sorted by increasing *p*-value.
(PDF)

S7 Table Purely spatial clusters. From left to right, the table shows, for each cluster, its identifier, its area (in km^2^), the number of surveyed sites falling inside the cluster and the number of surveys performed inside the area covered by the cluster during the whole period of study. The remaining columns are interpreted as in S6 Table.
(PDF)

S8 Table Purely temporal clusters. Each row corresponds to a subset of the whole dataset and reports the identifier of the cluster, the used data subset, the time interval of the most likely cluster, the total number of geolocated surveys in each data subset, and the number of surveys performed during the period of the most likely cluster. The remaining columns are interpreted as in S6 Table.
(PDF)

S9 Table Seasonal clusters. Each row corresponds to a subset of the whole dataset used in the analysis. From left to right, each row shows the identifier of the cluster, the used data subset, the period of the most likely seasonal cluster in the corresponding data subset, the total number of geolocated surveys in the data subset, and the number of surveys performed during the seasonal cluster (e.g., for non-clinical surveys, the number of surveys performed in the first three weeks of October was 403 out of 3617 surveys). The remaining columns are interpreted as in S6 Table.
(PDF)

S10 Table Prospective clusters. Each row corresponds to a simulation performed as if the current date were the date specified in the first column so that only data up to such time were available. From left to right, the table shows the simulation date, the identifier of the prospective cluster, the identifier of a corresponding cluster that was found by a retrospective analysis (see S6 Table the initial time of the prospective cluster, the area (in km^2^) of each cluster, the overall number of sites inside the area of the prospective cluster (independent of when they were surveyed) and the number of sites surveyed since the start date of the prospective cluster up to the current (simulation) date (#). The remaining columns are interpreted as in S6 Table.
(PDF)
