# Supplementary figures and images for: Environmental surveillance and spatio-temporal analysis of Legionella spp. in a region of northeastern Italy (2002–2017)
Source: PLoS One. 2019 Jul 9;14(7):e0218687. doi: 10.1371/journal.pone.0218687 (PMC6615612; doi:10.1371/journal.pone.0218687)

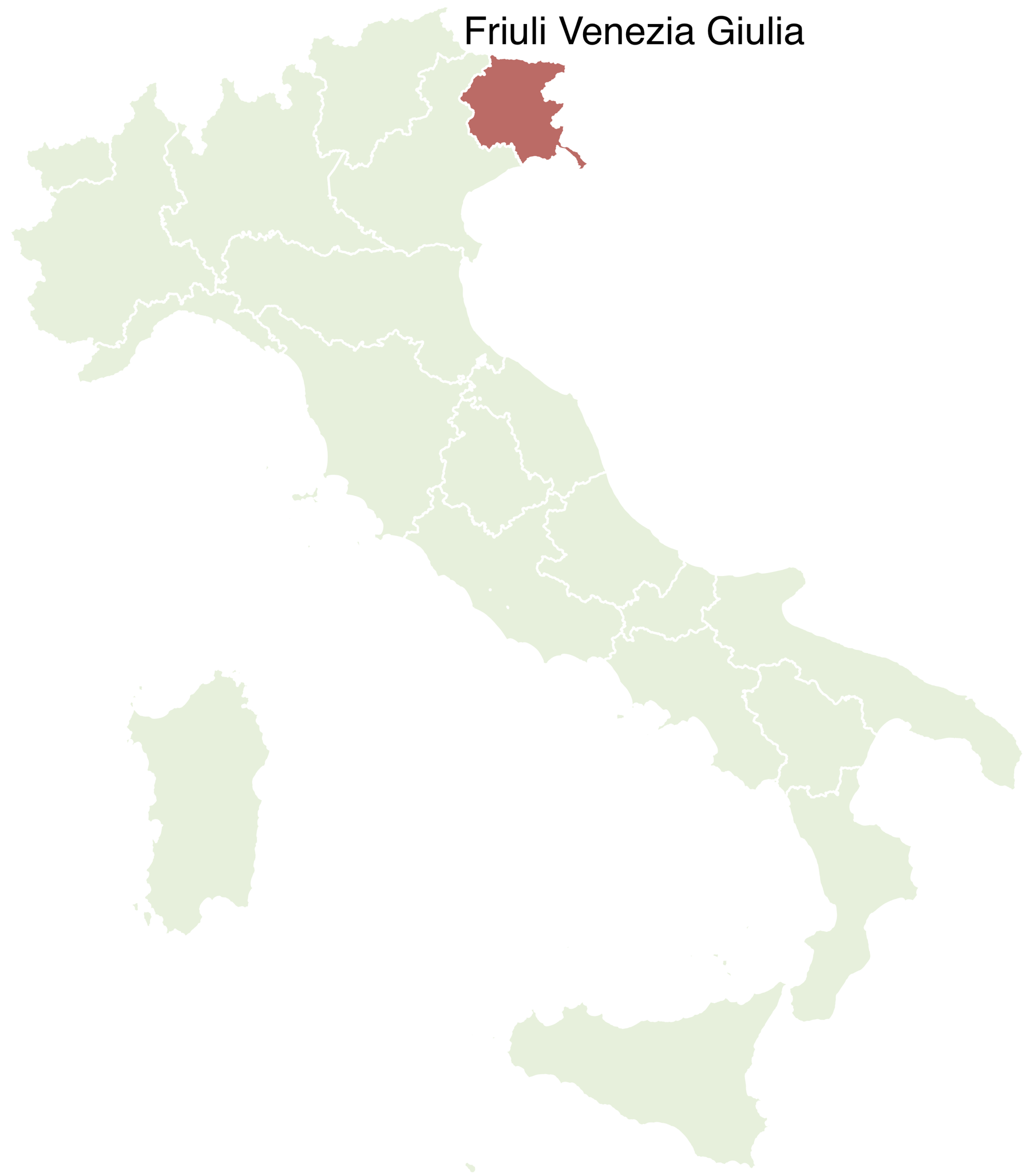

Supplement: S1 Fig — Friuli Venezia Giulia, the northeastern-most region of Italy, facing the Adriatic Sea and bordering Austria and Slovenia, has a population of approximately 1,200,000 inhabitants, a high quality of life and a touristic vocation. (TIF) [file pone.0218687.s001.tif]

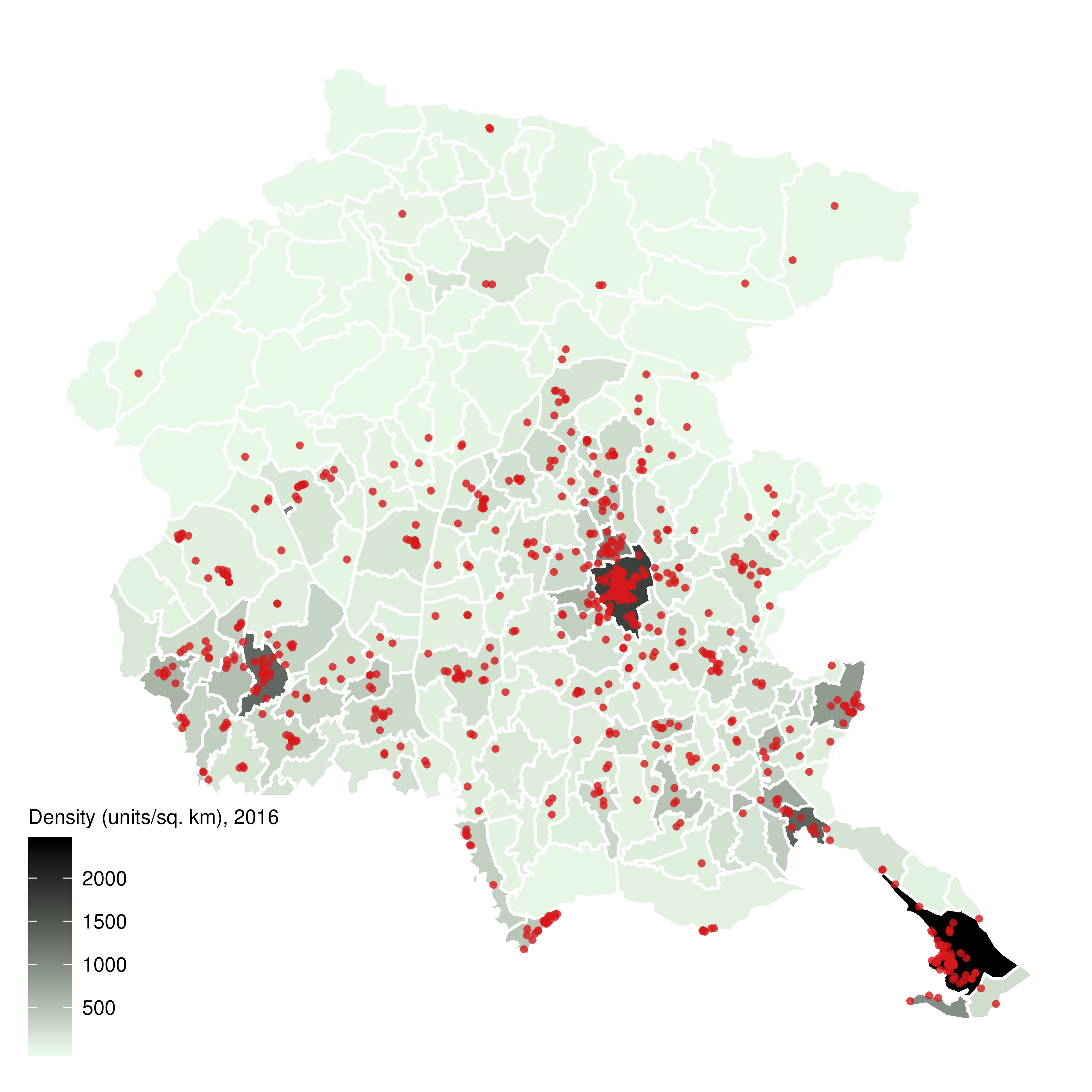

Supplement: S2 Fig — Each dot on the map corresponds to one of the 739 geolocalized sites where at least one survey was conducted during the period of study. The gradient represents the population density as of 2016 for each municipality in the region. The two main cities of Trieste and Udine are the most densely populated areas, while all of the northern part of Friuli Venezia Giulia is mountainous and sparsely populated. Consequently, the spatial distribution of surveys is highly nonuniform. (TIF) [file pone.0218687.s002.tif]

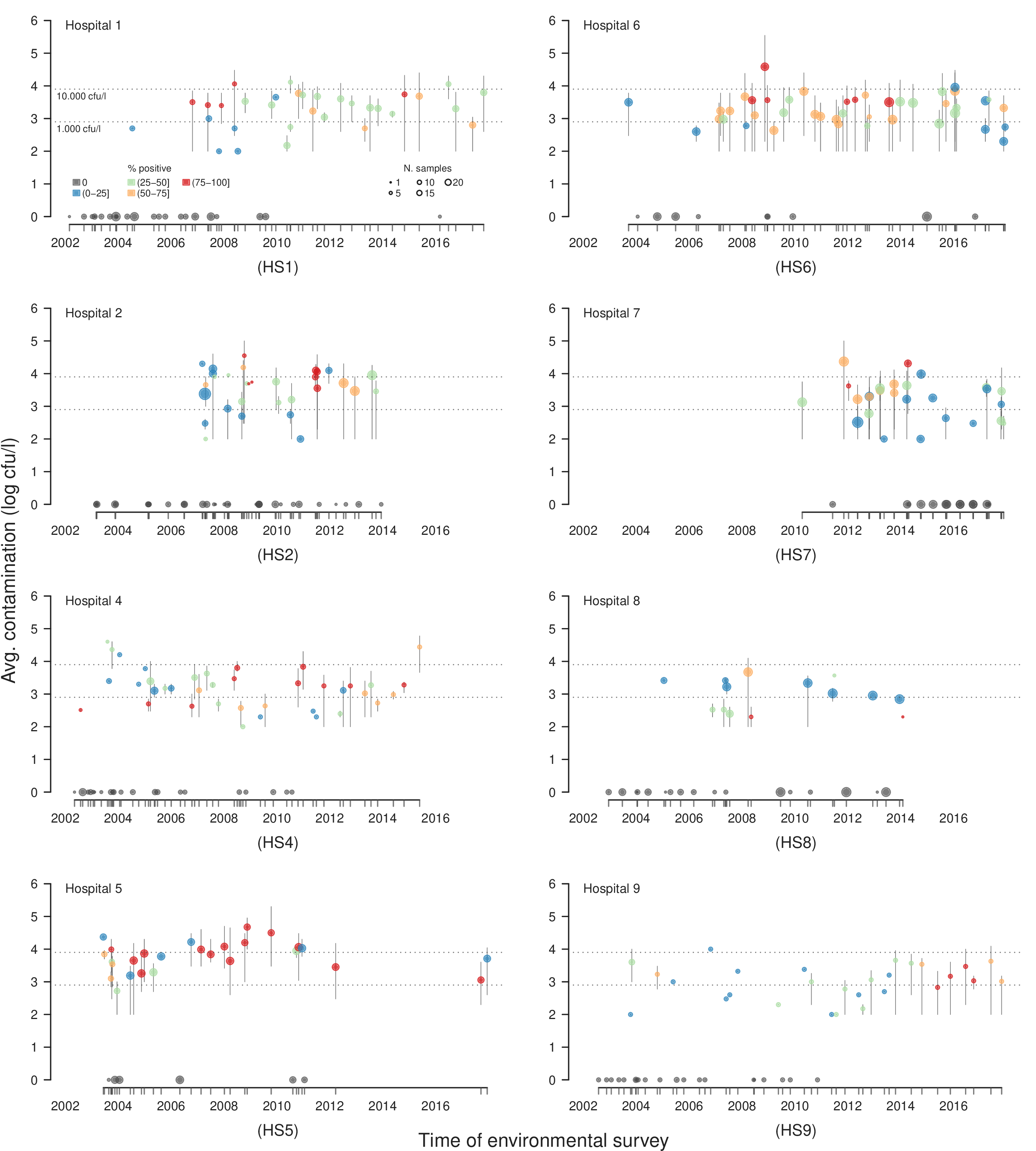

Supplement: S3 Fig — Each graph shows the data for the samples collected in one of the main hospitals of Friuli Venezia Giulia, Italy (the graph for Hospital 3 in the main article). Each data point represents the average positive contamination level of a survey (i.e., it is the average of the contamination levels of the positive samples of the survey), it is colored according to the percentage of positive samples of the survey, and its size is proportional to the total number of samples harvested during the survey (see the legend in the top-left figure). The vertical bars around each dot indicate the minimum and maximum positive contamination level found during each survey. The gray dots correspond to surveys in which all the samples resulted negative. (TIF) [file pone.0218687.s003.tif]

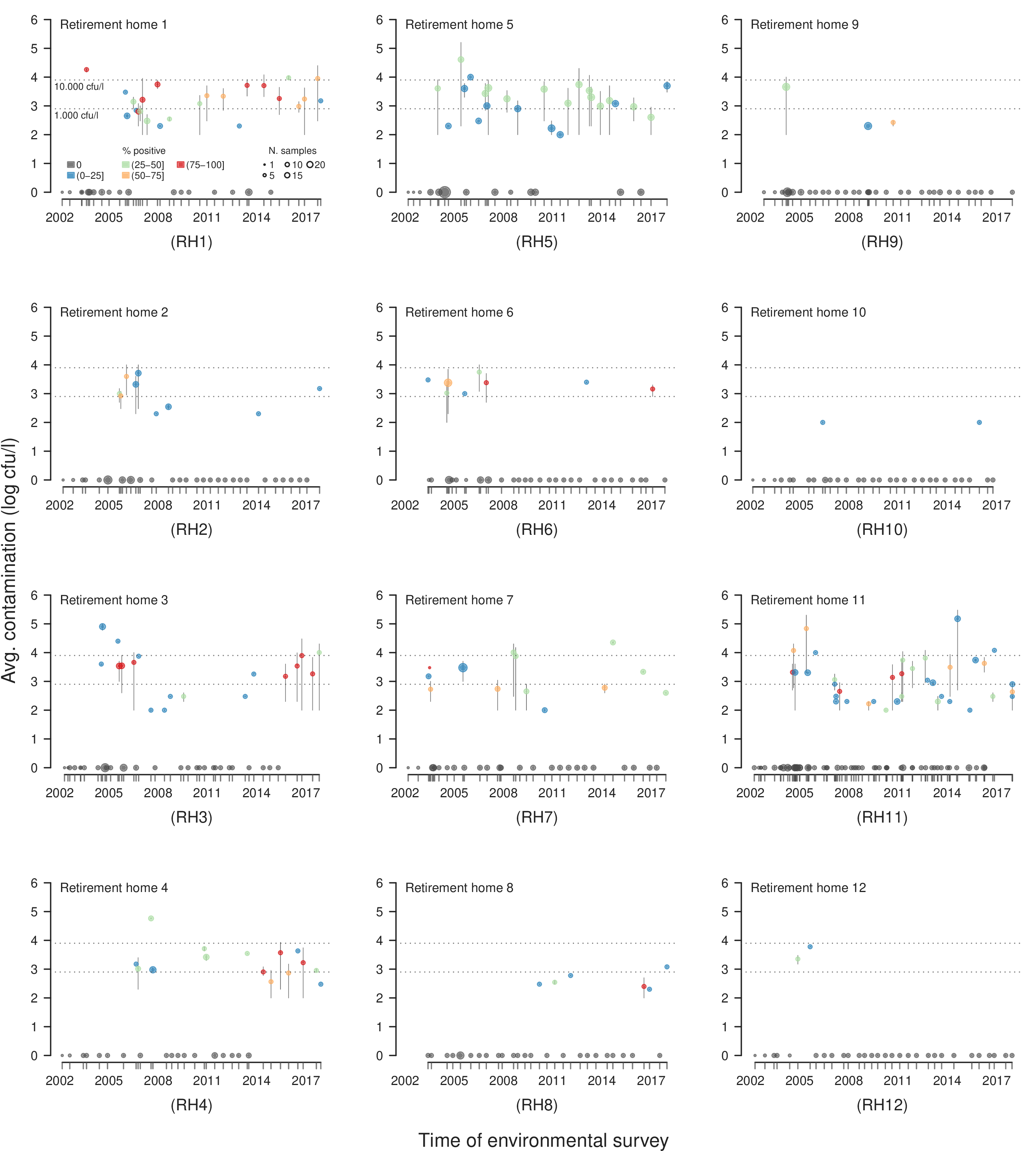

Supplement: S4 Fig — For interpreting the graphs, see Fig 2. (TIF) [file pone.0218687.s004.tif]

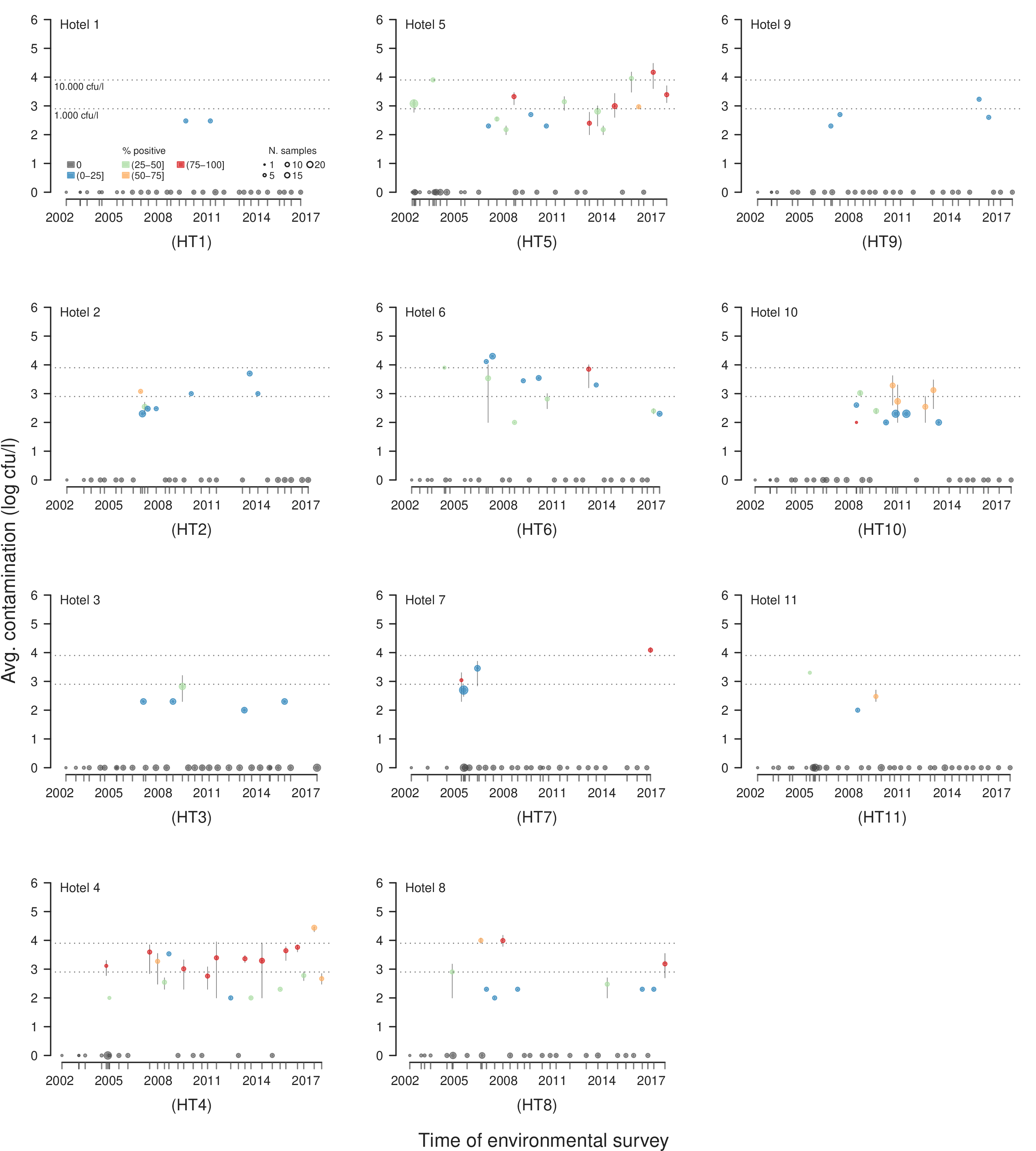

Supplement: S5 Fig — For interpreting the graphs, see Fig 2. (TIF) [file pone.0218687.s005.tif]

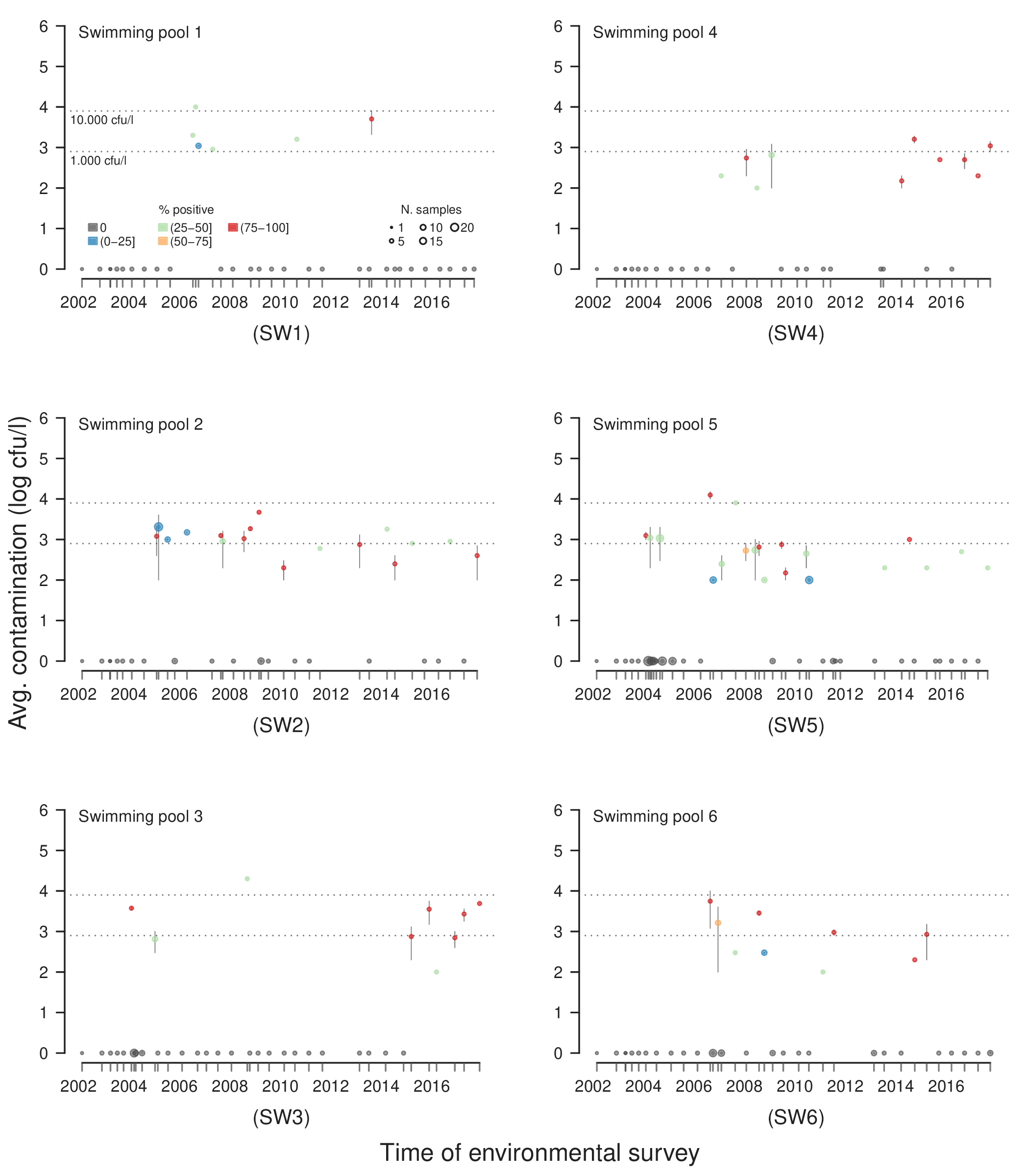

Supplement: S6 Fig — For interpreting the graphs, see Fig 2. (TIF) [file pone.0218687.s006.tif]

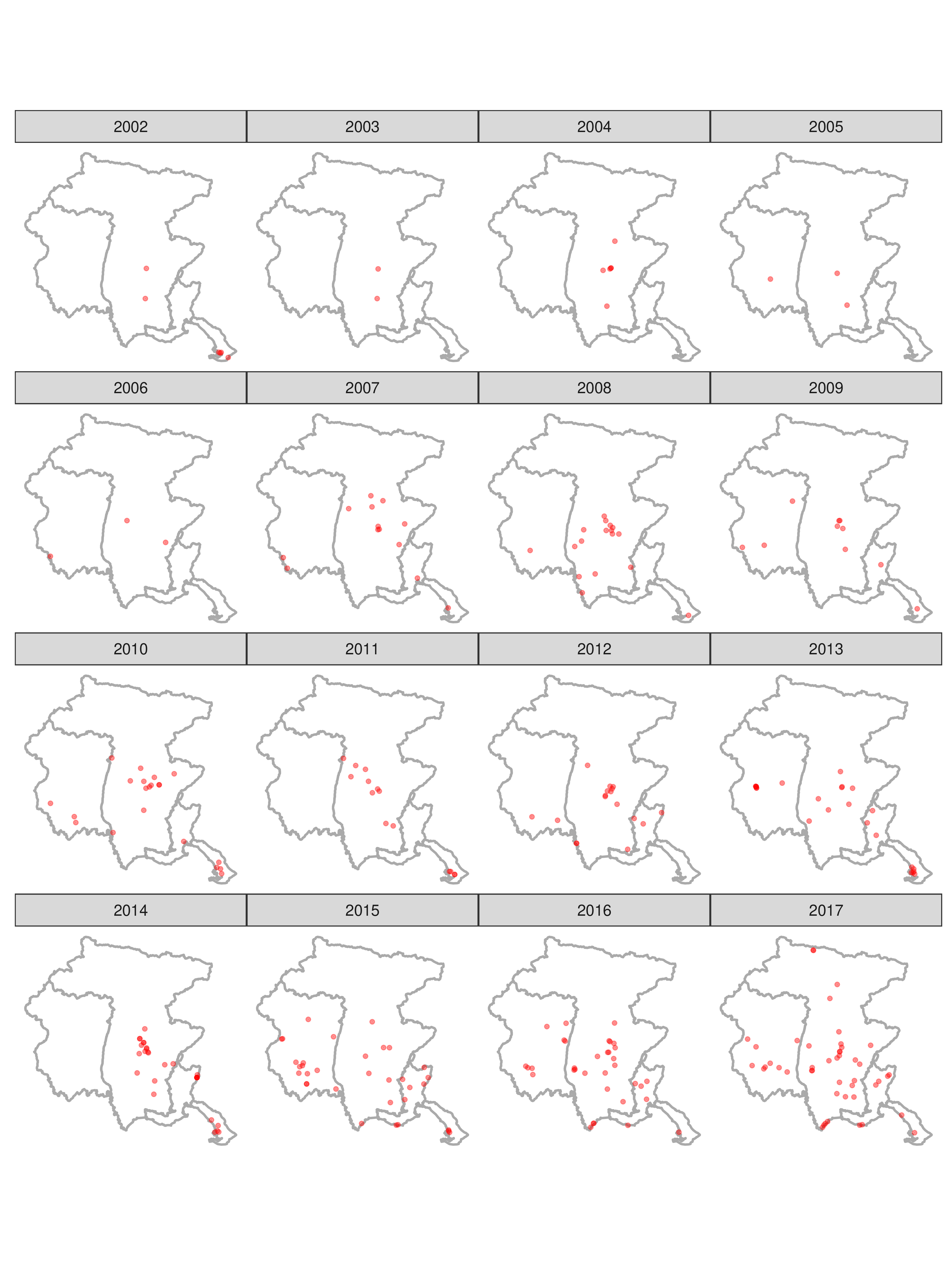

Supplement: S7 Fig — The figure shows the location of follow-up environmental surveys performed in Friuli Venezia Giulia after notifications of cases of legionellosis since 2002 to 2017. (TIF) [file pone.0218687.s007.tif]
