# Supplementary material for: Environmental surveillance and spatio-temporal analysis of Legionella spp. in a region of northeastern Italy (2002–2017)
Source: PLoS One. 2019 Jul 9;14(7):e0218687. doi: 10.1371/journal.pone.0218687 (PMC6615612; doi:10.1371/journal.pone.0218687)
Supplement: S1 Table — From left to right, the table shows, for each year, the overall number and percentage of positive surveys, and the number and percentage of low (100 ≤ CFUl−1 ≤ 1,000), medium (1,000 ≤ CFUl−1 ≤ 10,000) and high (>10,000 CFUl−1) risk surveys, respectively. A survey was considered “positive” when at least one sample associated to the survey tested positive. The risk level of each survey was determined based on the highest contamination level among the samples in the survey. (PDF) [file pone.0218687.s008.pdf]

Table S1: Yearly survey statistics. From left to right, the table shows, for each year, the total number of non-clinical surveys, the number and percentage of positive surveys, and the number and percentage of low ($100 \leq \mathrm{CFU\,l^{-1}} \leq 1{,}000$), medium ($1{,}000 \leq \mathrm{CFU\,l^{-1}} \leq 10{,}000$) and high ($>10{,}000\ \mathrm{CFU\,l^{-1}}$) risk surveys, respectively. A survey was considered "positive" when at least one sample associated to the survey tested positive. The risk level of each survey was determined based on the highest contamination level among the samples in the survey.

**Year**	**Surveys**	**Positive**		**Low risk**		**Medium risk**		**High risk**	
		**N.**	**Perc.**	**N.**	**Perc.**	**N.**	**Perc.**	**N.**	**Perc.**
2002	184	10	5.4%	5	50.0%	3	30.0%	2	20.0%
2003	300	34	11.3%	8	23.5%	19	55.9%	7	20.6%
2004	254	46	18.1%	8	17.4%	29	63.0%	9	19.6%
2005	260	59	22.7%	20	33.9%	34	57.6%	5	8.5%
2006	257	77	30.0%	24	31.2%	40	51.9%	13	16.9%
2007	288	132	45.8%	46	34.8%	60	45.5%	26	19.7%
2008	260	116	44.6%	39	33.6%	48	41.4%	29	25.0%
2009	262	83	31.7%	24	28.9%	46	55.4%	13	15.7%
2010	256	87	34.0%	33	37.9%	39	44.8%	15	17.2%
2011	233	72	30.9%	22	30.6%	35	48.6%	15	20.8%
2012	132	49	37.1%	14	28.6%	29	59.2%	6	12.2%
2013	226	79	35.0%	28	35.4%	40	50.6%	11	13.9%
2014	234	71	30.3%	27	38.0%	28	39.4%	16	22.5%
2015	180	56	31.1%	14	25.0%	27	48.2%	15	26.8%
2016	179	59	33.0%	25	42.4%	24	40.7%	10	16.9%
2017	143	68	47.6%	28	41.2%	27	39.7%	13	19.1%
