# Supplementary material for: Environmental surveillance and spatio-temporal analysis of Legionella spp. in a region of northeastern Italy (2002–2017)
Source: PLoS One. 2019 Jul 9;14(7):e0218687. doi: 10.1371/journal.pone.0218687 (PMC6615612; doi:10.1371/journal.pone.0218687)
Supplement: S2 Table — For each category, the total number of samples collected during the period of study and the number and percentage of positive samples are reported. The last three pairs of columns describe the distribution of positive samples across the corresponding risk levels: low (100 ≤ CFUl−1 ≤ 1,000), medium (1,000 ≤ CFUl−1 ≤ 10,000) and high (>10,000 CFUl−1) risk. Table rows are sorted in decreasing order with respect to the percentage of positive samples. (PDF) [file pone.0218687.s009.pdf]

Table S2: Samples by category of settings. For each category, the total number of samples collected during the period of study, the number of samples collected during follow-up surveys and the number and percentage of positive samples are reported. The last three pairs of columns describe the distribution of positive samples across the corresponding risk levels: low ($100 \leq \mathrm{CFU\,l^{-1}} \leq 1{,}000$), medium ($1{,}000 \leq \mathrm{CFU\,l^{-1}} \leq 10{,}000$) and high ($>10{,}000\ \mathrm{CFU\,l^{-1}}$) risk. Table rows are sorted in decreasing order with respect to the percentage of positive samples.

**Category**	**Total**	**Follow-up**	**Positive**		**Low risk**		**Medium risk**		**High risk**	
			**N.**	**Perc.**	**N.**	**Perc.**	**N.**	**Perc.**	**N.**	**Perc.**
Health	6447	76	1855	28.8%	863	46.5%	802	43.2%	190	10.2%
Military	121	16	26	21.5%	12	46.2%	10	38.5%	4	15.4%
Elderly	5300	11	841	15.9%	412	49.0%	355	42.2%	73	8.7%
Tourism	5074	174	781	15.4%	368	47.1%	297	38.0%	105	13.4%
Recreation	882	6	128	14.5%	76	59.4%	49	38.3%	2	1.6%
Private	1923	1923	257	13.4%	138	53.7%	94	36.6%	25	9.7%
School	99	0	11	11.1%	9	81.8%	0	0.0%	2	18.2%
Other	473	9	50	10.6%	33	66.0%	12	24.0%	5	10.0%
