# Supplementary material for: Environmental surveillance and spatio-temporal analysis of Legionella spp. in a region of northeastern Italy (2002–2017)
Source: PLoS One. 2019 Jul 9;14(7):e0218687. doi: 10.1371/journal.pone.0218687 (PMC6615612; doi:10.1371/journal.pone.0218687)
Supplement: S3 Table — Distribution of contamination levels for samples collected in cold water systems (or systems without hot water recirculation) and hot water circuits, respectively, across four risk levels: no risk (<100 CFUl−1), low risk (100 ≤ CFUl−1 ≤ 1,000), medium risk (1,000 ≤ CFUl−1 ≤ 10,000) and high risk (>10,000 CFUl−1). (PDF) [file pone.0218687.s010.pdf]

Table S3: Distribution of contamination levels for samples collected in cold water systems (or systems without hot water recirculation) and hot water circuits, respectively, across four risk levels: no risk (<100 CFU l^{-1}), low risk ($100 \leq$ CFU $l^{-1} \leq 1{,}000$), medium risk ($1{,}000 \leq$ CFU $l^{-1} \leq 10{,}000$) and high risk ($>10{,}000$ CFU l^{-1}).

Temperature	No risk	Low	Medium	High
Cold water circuit	86.1%	9.3%	3.5%	1.1%
Hot water circuit	83.3%	7.8%	7.1%	1.7%
