# Supplementary material for: Environmental surveillance and spatio-temporal analysis of Legionella spp. in a region of northeastern Italy (2002–2017)
Source: PLoS One. 2019 Jul 9;14(7):e0218687. doi: 10.1371/journal.pone.0218687 (PMC6615612; doi:10.1371/journal.pone.0218687)
Supplement: S4 Table — The table reports summary statistics about environmental surveys (top) and collected samples (bottom) from the main hospitals of Friuli Venezia Giulia. From left to right, the table shows the year, the number of surveyed hospitals, the overall number of conducted surveys (top) or overall number of collected samples (bottom) in each year, the number and percentage of positive surveys/samples, and the number and percentage of low (100 ≤ CFUl−1 ≤ 1,000), medium (1,000 ≤ CFUl−1 ≤ 10,000) and high (>10,000 CFUl−1) risk surveys/samples, respectively. A survey was considered “positive” when at least one sample associated to the survey tested positive. The risk level of each survey was determined based on the highest contamination level among the samples in the survey. (PDF) [file pone.0218687.s011.pdf]

Table S4: Temporal trends in the main hospitals. The table reports summary statistics about environmental surveys (top) and collected samples (bottom) from the main hospitals of Friuli Venezia Giulia. From left to right, the table shows the year, the number of surveyed hospitals, the overall number of conducted surveys (top) or overall number of collected samples (bottom) in each year, the number and percentage of positive surveys/samples, and the number and percentage of low ($100 \leq \mathrm{CFU\,l^{-1}} \leq 1{,}000$), medium ($1{,}000 \leq \mathrm{CFU\,l^{-1}} \leq 10{,}000$) and high ($>10{,}000\ \mathrm{CFU\,l^{-1}}$) risk surveys/samples, respectively. A survey was considered "positive" when at least one sample associated to the survey tested positive. The risk level of each survey was determined based on the highest contamination level among the samples in the survey.

Year	Hospitals	Total	Positive		Low risk		Medium risk		High risk	
			N.	Perc.	N.	Perc.	N.	Perc.	N.	Perc.
**Surveys**										
2002	5	12	1	8.3%	1	100.0%	0	0.0%	0	0.0%
2003	8	48	14	29.2%	3	21.4%	7	50.0%	4	28.6%
2004	7	25	8	32.0%	1	12.5%	4	50.0%	3	37.5%
2005	8	40	17	42.5%	7	41.2%	9	52.9%	1	5.9%
2006	8	30	16	53.3%	3	18.8%	10	62.5%	3	18.8%
2007	8	41	34	82.9%	9	26.5%	20	58.8%	5	14.7%
2008	8	48	37	77.1%	8	21.6%	15	40.5%	14	37.8%
2009	8	34	18	52.9%	4	22.2%	10	55.6%	4	22.2%
2010	9	34	26	76.5%	6	23.1%	12	46.2%	8	30.8%
2011	9	33	27	81.8%	5	18.5%	15	55.6%	7	25.9%
2012	9	27	23	85.2%	5	21.7%	14	60.9%	4	17.4%
2013	8	29	25	86.2%	6	24.0%	14	56.0%	5	20.0%
2014	7	21	17	81.0%	5	29.4%	7	41.2%	5	29.4%
2015	6	15	11	73.3%	1	9.1%	7	63.6%	3	27.3%
2016	5	20	14	70.0%	4	28.6%	6	42.9%	4	28.6%
2017	5	22	19	86.4%	7	36.8%	9	47.4%	3	15.8%
**Samples**										
2002	5	70	2	2.9%	2	100.0%	0	0.0%	0	0.0%
2003	8	298	39	13.1%	14	35.9%	20	51.3%	5	12.8%
2004	7	215	44	20.5%	10	22.7%	30	68.2%	4	9.1%
2005	8	307	42	13.7%	25	59.5%	16	38.1%	1	2.4%
2006	8	288	61	21.2%	38	62.3%	20	32.8%	3	4.9%
2007	8	387	132	34.1%	60	45.5%	65	49.2%	7	5.3%
2008	8	404	197	48.8%	75	38.1%	85	43.1%	37	18.8%
2009	8	334	112	33.5%	56	50.0%	49	43.8%	7	6.2%
2010	9	353	143	40.5%	66	46.2%	62	43.4%	15	10.5%
2011	9	350	162	46.3%	78	48.1%	65	40.1%	19	11.7%
2012	9	280	117	41.8%	64	54.7%	46	39.3%	7	6.0%
2013	8	319	128	40.1%	57	44.5%	61	47.7%	10	7.8%
2014	7	224	62	27.7%	21	33.9%	27	43.5%	14	22.6%
2015	6	185	58	31.4%	32	55.2%	22	37.9%	4	6.9%
2016	5	226	65	28.8%	30	46.2%	28	43.1%	7	10.8%
2017	6	237	63	26.6%	40	63.5%	20	31.7%	3	4.8%
