# Supplementary material for: Environmental surveillance and spatio-temporal analysis of Legionella spp. in a region of northeastern Italy (2002–2017)
Source: PLoS One. 2019 Jul 9;14(7):e0218687. doi: 10.1371/journal.pone.0218687 (PMC6615612; doi:10.1371/journal.pone.0218687)
Supplement: S5 Table — From left to right, the table shows the type of each unit, the number of distinct surveyed wards of each type, the overall number of collected samples for each unit type, the number and percentage of positive samples, and the number and percentage of low (100 ≤ CFUl−1 ≤ 1,000), medium (1,000 ≤ CFUl−1 ≤ 10,000) and high (>10,000 CFUl−1) risk samples, respectively. Data are sorted by decreasing percentage of positive samples. Only units in which at least 30 samples were collected during the period of study are shown. (PDF) [file pone.0218687.s012.pdf]

Table S5: Samples by hospital unit. From left to right, the table shows the type of each unit, the number of distinct surveyed wards of each type, the overall number of collected samples for each unit type, the number and percentage of positive samples, and the number and percentage of low ($100 \leq \mathrm{CFU\,l^{-1}} \leq 1{,}000$), medium ($1{,}000 \leq \mathrm{CFU\,l^{-1}} \leq 10{,}000$) and high ($>10{,}000\ \mathrm{CFU\,l^{-1}}$) risk samples, respectively. Data are sorted by decreasing percentage of positive samples. Only units in which at least 30 samples were collected during the period of study are shown.

**Unit**	**N. Wards**	**N. Samples**	**Positive**		**Low risk**		**Medium risk**		**High risk**	
			**N.**	**Perc.**	**N.**	**Perc.**	**N.**	**Perc.**	**N.**	**Perc.**
Neonatology	5	98	57	57.2%	28	49.1%	25	43.9%	4	7.0%
Obstetrics	10	236	123	52.1%	25	20.3%	77	62.6%	21	17.1%
Oncology	10	148	75	50.7%	41	54.7%	29	38.7%	5	6.7%
Haematology	2	119	56	47.1%	42	75.0%	14	25.0%	0	0.0%
Orthopaedics	14	184	75	40.8%	30	40.0%	37	49.3%	8	10.7%
Dialysis	11	72	28	38.9%	5	17.9%	14	50.0%	9	32.1%
Neurology	5	31	12	38.7%	4	33.3%	4	33.3%	4	33.3%
Surgery	17	394	119	30.2%	53	44.5%	53	44.5%	13	10.9%
Pediatrics	12	218	59	27.1%	32	54.2%	21	35.6%	6	10.2%
Day Hospital	6	33	8	24.2%	1	12.5%	7	87.5%	0	0.0%
Spinal unit	3	72	15	20.8%	10	66.7%	5	33.3%	0	0.0%
Intensive care	8	67	12	17.9%	3	25.0%	9	75.0%	0	0.0%
