# Supplementary material for: Environmental surveillance and spatio-temporal analysis of Legionella spp. in a region of northeastern Italy (2002–2017)
Source: PLoS One. 2019 Jul 9;14(7):e0218687. doi: 10.1371/journal.pone.0218687 (PMC6615612; doi:10.1371/journal.pone.0218687)
Supplement: S6 Table — From left to right, the table shows, for each cluster that reached statistical significance (p ≤ 0.05), its identifier, its temporal extension, its area (in km2), the overall number of sites falling inside the cluster’s area (indepedent of when they were surveyed), the number of environmental surveys conducted inside the area delimited by the cluster within the corresponding time frame, the ratio between observed and expected number of surveys for each risk level, the relative risk for each risk level (which represents how much more common surveys with a given risk level are compared to the baseline), the log-likelihood ratio and the p-value associated with each cluster. A risk level was associated to each survey based on the highest contamination level among the samples collected in each survey, as follows: no risk (<100 CFUl−1), low risk (100 ≤ CFUl−1 ≤ 1,000), medium risk (1,000 ≤ CFUl−1 ≤ 10,000) and high risk (>10,000 CFUl−1). Data are sorted by increasing p-value. (PDF) [file pone.0218687.s013.pdf]

Table S6: Spatio-temporal clusters of surveys. From left to right, the table shows, for each cluster that reached statistical significance ($p \leq 0.05$), its identifier, its temporal extension, its area (in km^2), the overall number of sites falling inside the cluster's area (independent of when they were surveyed), the number of environmental surveys conducted inside the area delimited by the cluster within the corresponding time frame, the ratio between observed and expected number of surveys for each risk level, the relative risk for each risk level (which represents how much more common surveys with a given risk level were compared to the baseline), the log-likelihood ratio and the p-value associated with each cluster. A risk level was associated to each survey based on the highest contamination level among the samples collected in each survey, as follows: no risk (<100 $\mathrm{CFU\,l^{-1}}$), low risk ($100 \leq \mathrm{CFU\,l^{-1}} \leq 1{,}000$), medium risk ($1{,}000 \leq \mathrm{CFU\,l^{-1}} \leq 10{,}000$) and high risk (>10,000 $\mathrm{CFU\,l^{-1}}$). Data are sorted by increasing p-value.

Id	Time frame	Area	Sites	Surveys	Obs/Exp	RR	LLR	P-value
ST1	2006/12/21 to 2009/3/20	33.2	44	39	4/27.19 (none), 6/3.90 (low), 29/7.90 (medium/high)	0.15 (none), 1.55 (low), 3.78 (medium/high)	33.0	0.001
ST2	2006/9/21 to 2008/12/20	35.9	10	13	0/9.06 (none), 1/1.30 (low), 2/1.90 (medium), 10/0.74 (high)	0.00 (none), 0.77 (low), 1.05 (medium), 14.22 (high)	26.2	0.001
ST3	2006/9/21 to 2008/12/20	2.2	7	27	4/18.83 (none), 23/8.17 (low/medium/high)	0.21 (none), 2.85 (low/medium/high)	17.7	0.002
ST4	2011/9/21 to 2013/12/20	69.1	5	13	0/9.06 (none), 2/1.30 (low), 11/2.63 (medium/high)	0.00 (none), 1.54 (low), 4.22 (medium/high)	16.7	0.004
ST5	2015/9/21 to 2017/12/20	1.6	14	16	1/11.16 (none), 4/1.60 (low), 6/2.34 (medium), 5/0.91 (high)	0.09 (none), 2.51 (low), 2.59 (medium), 5.63 (high)	15.5	0.011
ST6	2015/3/21 to 2017/6/20	1.1	3	9	0/7.18 (none/low), 9/1.82 (medium/high)	0.00 (none/low), 4.98 (medium/high)	14.4	0.033
