# Supplementary material for: Environmental surveillance and spatio-temporal analysis of Legionella spp. in a region of northeastern Italy (2002–2017)
Source: PLoS One. 2019 Jul 9;14(7):e0218687. doi: 10.1371/journal.pone.0218687 (PMC6615612; doi:10.1371/journal.pone.0218687)
Supplement: S7 Table — From left to right, the table shows, for each cluster, its identifier, its area (in km2), the number of surveyed sites falling inside the cluster and the number of surveys performed inside the area covered by the cluster during the whole period of study. The remaining columns are interpreted as in S6 Table. (PDF) [file pone.0218687.s014.pdf]

Table S7: Purely spatial clusters. From left to right, the table shows, for each cluster, its identifier, its area (in km^2), the number of surveyed sites falling inside the cluster and the number of surveys performed inside the area covered by the cluster during the whole period of study. The remaining columns are interpreted as in Table S6.

Id	Area	Sites	Surveys	Obs/Exp	RR	LLR	P-value
SP1	25.3	31	244	0.48 (none), 1.72 (low), 2.45 (medium/high)	0.46 (none), 1.81 (low), 2.73 (medium/high)	77.0	$<10^{-17}$
SP2	4.8	5	44	0.39 (none), 0.45 (low), 1.56 (medium), 8.02 (high)	0.39 (none), 0.45 (low), 1.57 (medium), 8.78 (high)	34.1	$1.4 \cdot 10^{-12}$
SP3	0	1	31	0.19 (none), 1.61 (low), 2.87 (medium), 5.12 (high)	0.18 (none), 1.62 (low), 2.92 (medium), 5.31 (high)	24.3	$1.9 \cdot 10^{-8}$
SP4	40.0	11	78	0.64 (none), 1.15 (low), 1.41 (medium), 4.37 (high)	0.64 (none), 1.16 (low), 1.42 (medium), 4.37 (high)	17.1	$2.0 \cdot 10^{-5}$
SP5	0	1	25	0.50 (none/low), 1.10 (medium), 7.76 (high)	0.50 (none/low), 1.10 (medium), 8.15 (high)	16.3	$4.8 \cdot 10^{-5}$
SP6	0.5	4	90	0.61 (none), 1.44 (low), 2.14 (medium/high)	0.60 (none), 1.46 (low), 2.20 (medium/high)	15.8	$7.5 \cdot 10^{-5}$
SP7	0.1	2	29	0.48 (none/low), 2.36 (medium), 4.87 (high)	0.47 (none/low), 2.39 (medium), 5.02 (high)	13.2	$9.0 \cdot 10^{-4}$
SP8	0.6	7	87	0.64 (none), 1.72 (low), 1.87 (medium/high)	0.64 (none), 1.75 (low), 1.91 (medium/high)	11.9	0.0032
SP9	1.1	3	81	0.67 (none), 1.60 (low), 1.86 (medium/high)	0.67 (none), 1.63 (low), 1.86 (medium/high)	9.4	0.045
