# Supplementary material for: Environmental surveillance and spatio-temporal analysis of Legionella spp. in a region of northeastern Italy (2002–2017)
Source: PLoS One. 2019 Jul 9;14(7):e0218687. doi: 10.1371/journal.pone.0218687 (PMC6615612; doi:10.1371/journal.pone.0218687)
Supplement: S8 Table — Each row corresponds to a subset of the whole dataset and reports the identifier of the cluster, the used data subset, the time interval of the most likely cluster, the total number of geolocated surveys in each data subset, and the number of surveys performed during the period of the most likely cluster. The remaining columns are interpreted as in S6 Table. (PDF) [file pone.0218687.s015.pdf]

Table S8: Purely temporal clusters. Each row corresponds to a subset of the whole dataset and reports the identifier of the cluster, the used data subset, the time interval of the most likely cluster, the total number of geolocated surveys in each data subset, and the number of surveys performed during the period of the most likely cluster. The remaining columns are interpreted as in Table S6.

Id	Data set	Most likely	Total	During	Obs/Exp	RR	LLR	P-value
T1	Non-clin. surveys	2006/10/3 to 2009/2/4	3617	629	338/438.58 (none), 227/154.77 (low/medium), 64/35.65 (high)	0.74 (none), 1.63 (low/medium), 2.16 (high)	44.9	0.001
T2	Clinical surveys	2017/2/27 to 2017/9/27	276	24	10/18.26 (none), 7/4.26 (low/medium), 7/1.48 (high)	0.53 (none), 1.75 (low/medium), 7.35 (high)	9.7	0.029
T3	Health	2006/8/10 to 2008/12/11	818	162	42/75.26 (none), 29/22.18 (low), 61/44.36 (medium), 30/20.20 (high)	0.50 (none), 1.41 (low), 1.52 (medium), 1.69 (high)	18.0	0.001
T4	Elderly	2006/10/11 to 2007/7/30	1114	71	31/51.69 (none), 31/16.19 (low/medium), 9/3.12 (high)	0.58 (none), 2.04 (low/medium), 3.31 (high)	15.0	0.001
T5	Tourism	2007/8/7 to 2007/11/8	1179	7	0/5.50 (none), 1/0.62 (low), 2/0.61 (medium), 4/0.28 (high)	0.00 (none), 1.63 (low), 3.35 (medium), 15.57 (high)	13.7	0.003
T6	Recreation	2006/8/23 to 2008/10/22	428	66	38/52.58 (none), 24/12.80 (low/medium), 4/0.62 (high)	0.69 (none), 2.23 (low/medium), +inf[1] (high)	14.9	0.001
