# Supplementary material for: Environmental surveillance and spatio-temporal analysis of Legionella spp. in a region of northeastern Italy (2002–2017)
Source: PLoS One. 2019 Jul 9;14(7):e0218687. doi: 10.1371/journal.pone.0218687 (PMC6615612; doi:10.1371/journal.pone.0218687)
Supplement: S9 Table — Each row corresponds to a subset of the whole dataset used in the analysis. From left to right, each row shows the identifier of the cluster, the used data subset, the period of the most likely seasonal cluster in the corresponding data subset, the total number of geolocated surveys in the data subset, and the number of surveys performed during the seasonal cluster (e.g., for non-clinical surveys, the number of surveys performed in the first three weeks of October was 403 out of 3617 surveys). The remaining columns are interpreted as in S6 Table. (PDF) [file pone.0218687.s016.pdf]

Table S9: Seasonal clusters. Each row corresponds to a subset of the whole dataset used in the analysis. From left to right, each row shows the identifier of the cluster, the used data subset, the period of the most likely seasonal cluster in the corresponding data subset, the number of surveys performed during the seasonal cluster over the total number of geolocated surveys in the data subset (e.g., for non-clinical surveys, the number of surveys performed in the first three weeks of October was 403 out of 3617 non-clinical surveys). The remaining columns are interpreted as in Table S6.

Id	Data set	Most likely cluster	Surveys/Total	Obs/Exp	RR	LLR	P-value
SE1	Non-clin. surveys	1 Oct to 23 Oct	403/3617	229/281.00 (none), 37/40.33 (low), 137/81.67 (medium/high)	0.80 (none), 0.91 (low), 1.83 (medium/high)	23.9	0.001
SE1	Clinical surveys	23 Jul to 17 Sep	97/319	62/72.98 (none), 10/8.51 (low), 25/15.51 (medium/high)	0.80 (none), 1.27 (low), 2.20 (medium/high)	5.25	0.116
SE1	Health	1 Oct to 12 Nov	154/818	49/71.54 (none), 16/21.09 (low), 89/61.37 (medium/high)	0.64 (none), 0.72 (low), 1.62 (medium/high)	12.6	0.001
SE1	Elderly	6 Aug to 15 Oct	269/1114	208/218.77 (none/low), 41/38.39 (medium), 20/11/93 (high)	0.94 (none/low), 1.09 (medium), 2.17 (high)	3.8	0.407
SE1	Tourism	20 Aug to 29 Oct	232/1179	182/202.68 (none/low), 30/20.07 (medium), 20/9.25 (high)	0.88 (none/low), 1.70 (medium), 3.02 (high)	10.5	0.002
SE1	Recreation	7 Aug to 20 Aug	19/428	8/15.14 (none), 3/1.86 (low), 7/1.82 (medium), 1/0.18 (high)	0.52 (none), 1.66 (low), 4.43 (medium), 7.18 (high)	8.0	0.015
