# Supplementary material for: Environmental surveillance and spatio-temporal analysis of Legionella spp. in a region of northeastern Italy (2002–2017)
Source: PLoS One. 2019 Jul 9;14(7):e0218687. doi: 10.1371/journal.pone.0218687 (PMC6615612; doi:10.1371/journal.pone.0218687)
Supplement: S10 Table — Each row corresponds to a simulation performed as if the current date were the date specified in the first column so that only data up to such time were available. From left to right, the table shows the simulation date, the identifier of the prospective cluster, the identifier of a corresponding cluster that was found by a retrospective analysis (see S6 Table the initial time of the prospective cluster, the area (in km2) of each cluster, the overall number of sites inside the area of the prospective cluster (independent of when they were surveyed) and the number of sites surveyed since the start date of the prospective cluster up to the current (simulation) date (#). The remaining columns are interpreted as in S6 Table. (PDF) [file pone.0218687.s017.pdf]

Table S10: Prospective clusters. Each row corresponds to a simulation performed as if the current date were the date specified in the first column so that only data up to such time were available. From left to right, the table shows the simulation date, the identifier of the prospective cluster, the identifier of a corresponding cluster that was found by a retrospective analysis (see Table S6), the initial time of the prospective cluster, the area (in km^2) of each cluster, the overall number of sites inside the area of the prospective cluster (independent of when they were surveyed) and the number of sites surveyed since the start date of the prospective cluster up to the current (simulation) date (#). The remaining columns are interpreted as in Table S6.

**Date**	**Id**	**Ref.**	**Start Date**	**Area**	**Sites**	**#**	**Obs/Exp**	**RR**	**LLR**	**P-value**
2007/11/26	P1	ST1	2006/12/12	33.2	44	14	0.08 (none/low), 5.82 (medium/high)	0.08 (none/low), 6.10 (medium/high)	20.7	0.001
2007/7/23	P2	ST2	2006/9/28	35.9	10	6	0.00 (none/low), 1.49 (medium), 23.79 (high)	0.00 (none), 1.50 (medium), 26.38 (high)	16.5	0.001
2006/11/27	P3	ST3	2006/10/3	29.8	54	5	0.00 (none), 4.29 (low), 6.27 (medium/high)	0.00 (none), 4.35 (low), 6.42 (medium/high)	8.9	0.047
2017/3/3	P4	ST5	2015/4/17	3.1	14	14	0.11 (none), 2.01 (low/medium), 5.08 (high)	0.11 (none), 2.02 (low/medium), 5.21 (high)	0.008	
2016/10/28	P5	ST6	2015/5/15	1.1	3	7	0.00 (none/low), 4.22 (medium/high)	0.00 (none/low), 4.26 (medium/high)	10.1	0.05
